# The effect of tanshinones on cognitive impairments in animal models of Alzheimer’s disease: a systematic review and meta-analysis

**DOI:** 10.3389/fphar.2025.1529327

**Published:** 2025-02-27

**Authors:** Shuwei Wang, Jinsha Yang, Wenbin Zheng, Serena Zhang, Dayong Zhong

**Affiliations:** ^1^ Hospital of Chengdu University of Traditional Chinese Medicine, Chengdu, China; ^2^ Sichuan Province Famous Traditional Chinese Medicine, Chengdu, China; ^3^ Enloe Magnet High School, Raleigh, NC, United States; ^4^ Third Veterans Hospital of Sichuan Province, Chengdu, China

**Keywords:** tanshinones, Alzheimer’s disease, cognitive impairment, animal models, meta-analysis

## Abstract

**Background:**

Alzheimer’s disease (AD) is an age-related neurological illness that poses a significant hazard to human health. A fat-soluble compound called tanshinones was isolated from Danshen, a traditional Chinese herb. Recent years have seen reports of clinical trials examining the effects of tanshinones on cognitive impairment among individuals with AD, as well as the publication of pertinent basic research. Tanshinones are not yet commonly utilized in the therapeutic treatment of AD, and the effectiveness of tanshinones as a treatment program for AD is not yet adequately supported by evidence. To assess the impact of tanshinones on cognitive impairment in experimental rodent models of AD, we carried out a systematic review in this work.

**Method:**

All relevant studies on the usage of tanshinones in AD model animals published in PubMed, Cochrane Library, Web of Science, EMBASE, Chinese Biomedicine Database, and China National Knowledge Infrastructure before 8 September 2024, were systematically retrieved. To assess the methodological quality, the CAMARADES checklist was used. Meta-analysis was calculated and graphed in the Stata 14.0 software. For each outcome in every study, the standard mean difference (SMD) and the 95% confidence interval (CI) of each effect size were calculated.

**Results:**

Fourteen studies were included in this study. Compared with the AD model group without tanshinones intervention, tanshinones significantly reduced the number of escape latency [SMD = −2.082, 95% CI = (−2.481, −1.683), p < 0.001]. Tanshinones also increased the times of platform crossing [SMD = 1.464, 95% CI = (1.183, 1.744), p < 0.001] and time in target quadrants [SMD = 2.703, 95% CI = (2.132, 3.275), p < 0.001].

**Conclusion:**

Tanshinones are thought to have positive effects on cognitive impairment in rodent models of AD, according to the findings of this study. However, the level of quality of the included research may have an impact on the accuracy of positive outcomes. Thus, more high-quality randomized controlled animal studies are required to guide future scientific and clinical research.

**Systematic Review Registration:**

identifier CRD42024557980

## 1 Introduction

Currently, 6.9 million Americans age 65 and older are estimated to have Alzheimer’s disease (AD). If no medical advances are made to prevent or treat AD, this figure may increase to 13.8 million by 2060 (Author Anonymous, 2024). The prevalence of AD patients has rapidly increased as a result of an aging population. According to recent estimates, by 2050, the prevalence of AD is expected to quadruple globally and double in Europe (Scheltens et al., 2021). In the world, it turns into a public health dilemma, and the direct costs of AD to society are substantial. Cognitive impairment is one of the most important disabilities linked to AD (Bolognin et al., 2012). This handicap has a difficult-to-quantify personal and financial cost and interferes with relationships, employment, leisure, and everyday life activities.

One of the main characteristics of AD is neurodegeneration, which is accompanied by beta-amyloid and tau buildup. Two of the various brain alterations linked to AD are the buildup of an aberrant version of the protein tau (called tau tangles) inside neurons and the aggregation of the protein fragment beta-amyloid into clumps (called beta-amyloid plaques) outside neurons. Tau and beta-amyloid play distinct functions in AD. Plaques and smaller beta-amyloid accumulations can harm neurons by disrupting synaptic connections between neurons. Tau tangles damage connections between neurons while obstructing the passage of nutrients and other chemicals necessary for neurons’ survival and regular operation inside neurons (Author Anonymous, 2024). There aren’t many treatment options available to stop or lessen AD-related cognitive deficits.

“Shennong’s Herbal Classic” was the first source of information about Danshen, a traditional Chinese medicine derived from the dried root and rhizome of Salvia miltiorrhizae Bunge, a native Chinese perennial plant of the Labiatae family. Danshen, which is associated with the liver and heart meridians, has a slightly chilly temperament and a bitter flavor. Traditional Chinese medicine claims that Danshen is beneficial for removing vexation and clearing the heart, as well as for promoting blood circulation, removing blood stasis, cooling the blood, getting rid of carbuncles, easing menstruation, and reducing pain (Pharmacopoeia of the People’s Republic of China, 2020). Additionally, Danshen is frequently used in a variety of dose forms to treat cerebrovascular and cardiovascular disorders (Writing Group of Recommendations of Expert Panel from Chinese Geriatrics Society on the Clinical Use of Compound Danshen Dripping Pills, 2017). The most significant pharmacologically active components in Danshen are tanshinones, including Dihydrotanshinone I, Tanshinone IIA, Tanshinone I, and Cryptotanshinone (Nwafor et al., 2021). Tanshinones have been shown in recent research to exhibit protective effects against AD by preventing the processing of amyloid precursor proteins, tau hyperphosphorylation, mitochondrial dysfunction, and aberrant autophagy (Chong et al., 2019).

Tanshinones may considerably enhance cognitive performance and patients’ quality of life, according to a few clinical investigations (Zheng et al., 2023; Guo et al., 2020). The mechanism by which tanshinones treat AD has been the subject of an increasing amount of preclinical research in recent years. Numerous studies have confirmed that tanshinones can enhance the cognitive function of AD model animals, offering both theoretical and experimental support for the potential clinical application of tanshinones in the treatment of AD. According to recent research, tanshinone IIA increases levels of rats’ synapse-associated proteins synaptophysin (SYP) and postsynaptic dense substance 95 (PSD-95) (Jia et al., 2023), while additionally decreasing the increase in cyclooxygenase-2 (COX-2) expression and prostaglandin E2 (PGE2) secretion caused by Aβ through the inactivation of the nuclear transcription factor kappa (NF-κB) pathway (Geng et al., 2019).

Meta-analyses of animal research have been shown to be beneficial, frequently offering fresh and significant insights from previously published animal studies. They are able to recommend ways to improve animal management, pinpoint locations for more animal trials, and methodically assess the effectiveness, assist in resolving discrepancies between preclinical studies, and provide a basis for future clinical research (Hooijmans et al., 2014; Luís et al., 2023). Nevertheless, no comprehensive evaluation has yet to provide proof of tashinones’ ability to lessen cognitive impairment in animals used as models for AD.

To bridge the research gaps mentioned above, this study aims to: (1) Update current research and perform a systematic review and meta-analysis of the efficacy of tanshinones in treating AD model animals. (2) Address concerns about research rigor, reliability, and confounders by limiting the studies to animal experiments and excluding studies in which tanshinones were combined with other interventions. (3) Conduct a thorough investigation of experimental animal models, tanshinone type and dosage, administration route, and duration of treatment. A preliminary screening of more effective tanshinones therapeutic regimens can be conducted by observing whether animal models of AD will have a positive response after tanshinones intervention.

## 2 Materials and methods

The PRISMA Extension for Chinese Herbal Medicines 2020 (PRISMA-CHM, 2020) (Zhang et al., 2020) and the Preferred Reporting Items for Systematic Reviews and Meta-Analysis (PRISMA) (Moher et al., 2015) served as the foundation for the meta-analysis. Since all of the study’s data came from publicly accessible sources, an ethical review was not required. The International Prospective Register of Systematic Reviews (PROSPERO) now has the protocol for this meta-analysis registered (Registration number CRD42024557980).

### 2.1 Search strategy

In order to gather experimental data on the impact of tanshinones in animal models of AD, two researchers with the same education independently searched PubMed, Web of Science, the Cochrane Library, EMBASE, China National Knowledge Infrastructure, and Chinese Biomedicine Database. A third researcher resolved any disagreements. All searches were restricted to works of literature released before 8 September 2024. “Tanshinone” or “Salvia miltiorrhiza” and “Alzheimer’s” or “Alzheimer” or “dementia” or “cognition” or “cognitive” were the subject phrases. All searches and downloads were finished on 9 September 2024, to prevent any prejudice that might arise from later database changes. Supplement Material S1 contains a detailed search strategy.

### 2.2 Study selection and eligibility criteria

After removing duplicates, two researchers (Shuwei Wang and Jinsha Yang) with the same training independently reviewed the titles and abstracts based on the inclusion and exclusion criteria for preliminary screening. The two researchers then read the entire text of the pertinent literature to determine whether or not to include it. If there were conflicting views, they were either discussed or sent to Dayong Zhong, the third researcher, for evaluation.

The following were the predetermined inclusion criteria: (1) Experimental AD model was induced in rats or mice, which served as the research subjects; (2) The AD treatment groups received only tanshinone; (3) The AD model control groups were blank or received saline; (4) The Morris water maze (MWM) was utilized to measure cognitive function; (5) Studies involving animal models of AD; (6) Studies written in either Chinese or English.

The following were the predetermined exclusion criteria (1) Other types of animals (e.g., sheep, cats, and dogs) were used; (2) In addition to tanshinones, the treatment group received another neuroprotective agent or Chinese traditional medicine; (3) Combination treatments that involved physical therapy or exercise; (4) No AD model control group was used; (5) The evaluation of treatment efficacy was limited to biochemical or physiological outcomes; (6) Reviews, conferences, case reports, and clinical trial studies; (7) Research data was either incomplete, incorrect, or impossible to extract; (8) Research data for the same population was published repeatedly.

### 2.3 Data extraction

Two researchers extracted information about the studies. The main contents of data extraction were followed: (1) General information, including the title of the study, the name of the first author, the year of publication, the type of AD model, animal species, and weight; (2) Intervention measures, including drug name, dosage, administration mode, and duration; (3) Data and indicators related to the outcome; (4) GetData Graph Digitizer software was used to extract data from graphs. For cases where the data were missing, the researchers contacted the authors and requested the additional information. If the required data were not available, then the study was excluded from the analysis.

In every study that was part of the analysis, MWM was employed to evaluate cognition. Only the final assessment, if cognition was evaluated multiple times during the study, was analyzed.

### 2.4 Quality assessment

A checklist that was slightly altered from the Collaborative Approach to Meta-Analysis and Review of Animal Data from Experimental Studies (CAMARADES) was used to evaluate the studies’ methodological quality. In the altered CAMARADES, blinded induction of AD (allocation concealment) was replaced by randomization of participants into treatment groups (Sheng et al., 2015), since blinded induction is more suitable for clinical trial studies, and it is difficult to achieve blinding in animal experimental model induction and allocation. One score was tallied for written evidence of each of the following criteria: (1) peer-reviewed publication; (2) randomization of subjects into treatment groups; (3) assessment of dose-response relationship; (4) blind assessment of behavioral outcomes; (5) monitoring of physiological parameters, such as body temperature; (6) calculation of the sample size necessary to achieve sufficient power; (7) statement of compliance with animal welfare regulations; (8) avoidance of anesthetic agents with marked intrinsic neuroprotective properties (e.g., ketamine); (9) statement of potential conflicts of interest (10) use of a suitable animal model. Each study was given a quality score ranging from zero to ten. The greater the score, the higher the quality of the article.

The study quality also was assessed with secondary criteria as the following criteria described (Hooijmans et al., 2012). These criteria included study characteristics such as the age, species, and sex of the animals used; the duration of supplementation; and the dose(s) of tanshinone. These criteria also included an assessment of the internal validity of the study, i.e., performance bias (differences in care provided?); exclusion bias (differences in withdrawal from studies?); detection bias (differences in outcome measurements?); and selection bias (differences in allocation to comparison groups?), as well as an assessment of the external validity of the population, intervention, and outcome. Each study was given a quality score ranging from zero to twenty-one. The greater the score, the higher the quality of the article. Two researchers (Shuwei Wang and Jinsha Yang) were responsible for completing it.

### 2.5 Data analysis

All the data were analyzed using the Stata14.0 software. The standardized mean difference (SMD) with 95% confidence intervals (CI) was used for continuous variables to pool the results. The Higgins I^2^ statistic was used to measure the inconsistency among the results of the included studies, making it possible to classify the heterogeneity as low (25%), moderate (50%), and high (75%) (Higgins et al., 2003). Either a fixed effect model or a random effect model was applied to synthesize the data, depending on the heterogeneity test. The fixed effect model was used when the heterogeneity test results in the included studies were P > 0.1 and I^2^ < 50%; the random effect model was utilized when P < 0.1 and I^2^ > 50%. Additionally, the sensitivity analysis was performed by eliminating each study separately in order to evaluate the results’ stability. To account for heterogeneity and determine whether treatment effects differ between subgroups based on the following criteria: (1) experimental animal models; (2) tanshinone type and dosage; (3) administration route; and (4) duration of the treatment. These subgroup analyses were selected based on previous main sources of heterogeneity in meta-analysis studies in animal experiments (Luís et al., 2023; Sheng et al., 2015).

Publication bias was assessed by funnel plot (Light et al., 1994) and Egger’s regression test (Egger et al., 1997), and the reliability of the results was tested by the trim and fill technique (Duval and Tweedie, 2000).

## 3 Results

### 3.1 Selection process

**FIGURE 1:**
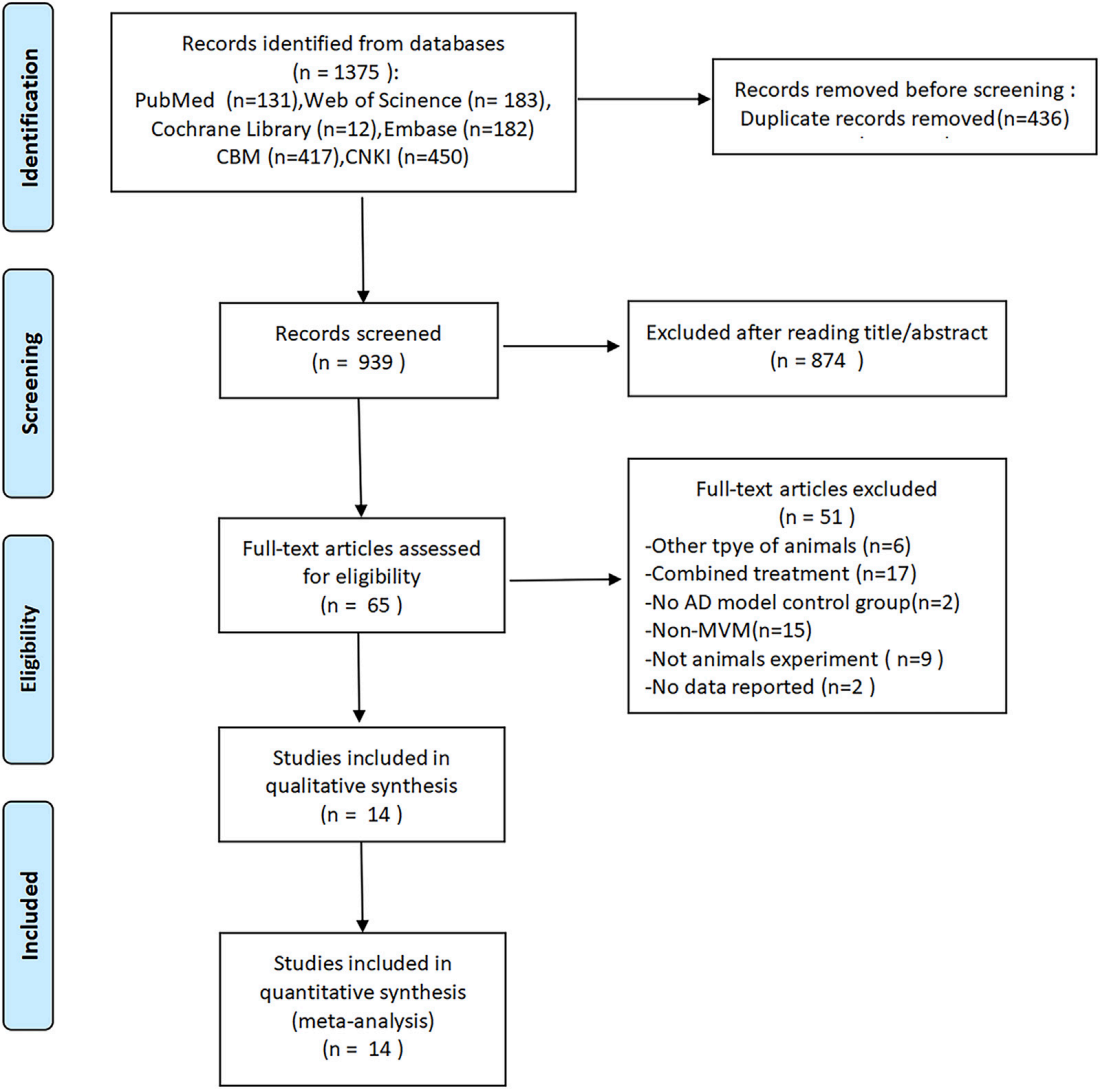
Flow chart depicting the literature screening process.

1,375 records have been identified in the database searches (Figure 1). 939 records remained after removing the duplicate records. 874 records that did not fulfill the inclusion criteria were eliminated after screening title and abstract; for example, most of them were removed because there were either no tanshinones or no animal model for AD. Subsequently, 14 studies met the inclusion criteria after the full-text analysis of the remaining 65 studies. Thus, this meta-analysis is based on 14 studies, comprising 26 platform crossing times comparisons, 28 time in target quadrant comparisons, and 37 escape latency comparisons. Half of the included researches were published in the last 10 years (2014–2024), with the earliest included study being published in 2009, suggesting that there has been a recent surge in interest in tanshinones’ impact on AD.

### 3.2 Study characteristics

Of the total 14 included studies (Table 1) (He Y. Y. et al., 2020; Liao, 2020; Liao et al., 2019; Mei et al., 2010; Wan, 2022; Sheraz, 2019; Fang et al., 2021; Mei et al., 2009; Ma et al., 2022; Xiang et al., 2024; He Y. et al., 2020; Ding et al., 2020; Peng et al., 2022; Liu et al., 2016), half of them were published in academic journals in English, while 7 were published in Chinese (He Y. Y. et al., 2020; Liao et al., 2019; Mei et al., 2010; Fang et al., 2021; Liao, 2020; Wan, 2022; Sheraz, 2019), including 3 academic dissertations (Liao, 2020; Wan, 2022; Sheraz, 2019), which have been peer-reviewed and published. The most commonly utilized animal model of AD was the APP/PS1 double transgenic model (Mei et al., 2010; Wan, 2022; Mei et al., 2009; Ma et al., 2022; He Y. et al., 2020; Ding et al., 2020; Peng et al., 2022). Of the 14 studies, 3 used non-transgenic mice (He Y. Y. et al., 2020; Fang et al., 2021; Liu et al., 2016), 4 used Sprague Dawley rats (Liao, 2020; Liao et al., 2019; Sheraz, 2019; Xiang et al., 2024), and 7 used APP/PS1 double transgenic mice (Mei et al., 2010; Wan, 2022; Mei et al., 2009; Ma et al., 2022; He Y. et al., 2020; Ding et al., 2020; Peng et al., 2022). 11 studies used male animals only (He Y. Y. et al., 2020; Liao, 2020; Liao et al., 2019; Wan, 2022; Sheraz, 2019; Fang et al., 2021; Xiang et al., 2024; He Y. et al., 2020; Ding et al., 2020; Peng et al., 2022; Liu et al., 2016), occupying the vast majority. Equal numbers of male and female animals were utilized in one study (Mei et al., 2010). The sex of the animals utilized was not reported in the rest of studies (Mei et al., 2009; Ma et al., 2022). Regarding the route of administration, experimenters tended to utilize intragastric administration, representing 8 studies (He Y. Y. et al., 2020; Liao, 2020; Liao et al., 2019; Mei et al., 2010; Wan, 2022; Sheraz, 2019; Xiang et al., 2024; Liu et al., 2016). 5 studies (Fang et al., 2021; Ma et al., 2022; He Y. et al., 2020; Ding et al., 2020; Peng et al., 2022) used intraperitoneal injection, while only one study (Mei et al., 2009) used oral administration. Across all studies, as experimental treatments, 1, 5, 10, 15, 20, 25, 30, 40, 45, 50, 75, or 80 mg/kg/day of either tanshinone IIA or cryptotanshinone were given. The MWM was employed in every study to evaluate cognitive function.

**TABLE 1:** Characteristics of included studies.

Study	Sex, strain, species	AD model	Main experimental groups (drugs dosage, route)	Intervention time	Duration of treatment
He et al. (2020)	Male Kunming mice	STZ model	C: CMC-Na, i.g. E: Tan IIA, 20/40/80 mg/kg, i.g.	Immediately after injury	28 days
Liao (2020)	Male SD rats	CCL_2_ model	C: NR E: Tan IIA, 25/50/75 mg/kg, i.g.	3 days before CCL_2_ injury	3 days
Liao et al. (2019)	Male SD rats	CCL_2_ model	C: NR E: Tan IIA, 25/50/75 mg/kg, i.g.	3 days before CCL_2_ injury	3 days
Mei et al. (2010)	Male and female APP/PS1 mice	APP/PS1 model	C: CMC-Na, i.g. E: CTS, 5/15/45 mg/kg, i.g.	3 months of age	16 weeks
Wan (2022)	Male APP/PS1 mice	APP/PS1 model	C: NR E: Tan IIA, 10/20 mg/kg, i.g.	6 months of age	8 weeks
Sheraz (2019)	Male SD rats	Aβ1-42 model	C: saline, i.g. E: Tan IIA, 20/40/80 mg/kg, i.g.	Immediately after injury	30 days
Fang et al. (2021)	Male ICR mice	LPS model	C: saline, i.p. E: Tan IIA, 1/5/10 mg/kg, i.p.	1 day after LPS injected	7 weeks
Mei et al. (2009)	APP/PS1 mice	APP/PS1 model	C: NR E: CTS, 5/15/30 mg/kg, p.o.	3 months of age	16 weeks
Ma et al. (2022)	APP/PS1 mice	APP/PS1 model	C: saline, i.p. E: Tan IIA, 10/20 mg/kg, i.p.	12 months of age	8 weeks
Xiang et al. (2024)	Male SD rats	STZ model	C: NR E: Tan IIA, 20/40/80 mg/kg, i.g.	Immediately after injury	24 days
He et al. (2020)	Male APP/PS1 mice	APP/PS1 model	C: saline, i.p. E: Tan IIA, 10/30 mg/kg, i.p.	6 months of age	30 days
Ding et al. (2020)	Male APP/PS1 mice	APP/PS1 model	C: saline, i.p. E: Tan IIA, 5/20 mg/kg, i.p.	6 months of age	30 days
Peng et al. (2022)	Male APP/PS1 mice	APP/PS1 model	C: saline, i.p. E: Tan IIA, 15/30 mg/kg, i.p.	5 months of age	30 days
Liu et al. (2016)	Male Swiss albino mice	STZ model	C: CMC-Na, i.g. E: Tan IIA, 20/40/80 mg/kg, i.g.	1 h before STZ injectd	28 days

Abbreviations: LPS, lipopolysaccharide; SD, sprague dawley; CCL_2_, CC, chemokine ligand 2; STZ, streptozotocin; C, control group; E, experimental group; NR, no report; CMC-Na, sodium carboxymethyl cellulose; Tan IIA, tanshinone IIA; CTS, cryptotanshinone; i. g., intragastrically; i. p., intraperitoneally; p. o., per os.

The primary clinical sign of AD is cognitive impairment, and the most crucial way to track AD mouse models of cognitive function is to evaluate behavioral functions related to memory. MWM is a well-known experiment for assessing learning, memory, and cognitive function in animal models of AD (Lissner et al., 2021). It involves forcing experimental animals (mice and rats) to swim and learn to locate a hidden platform submerged in water. The escape delay in the spatial exploration experiment is a measure of acquisition memory, whereas platform crossing times and time in the target quadrant are used to assess the retention memory of mice, which shows their capacity to retain and retrieve memories (Othman et al., 2022).

### 3.3 Methodology quality of studies

**FIGURE 2:**
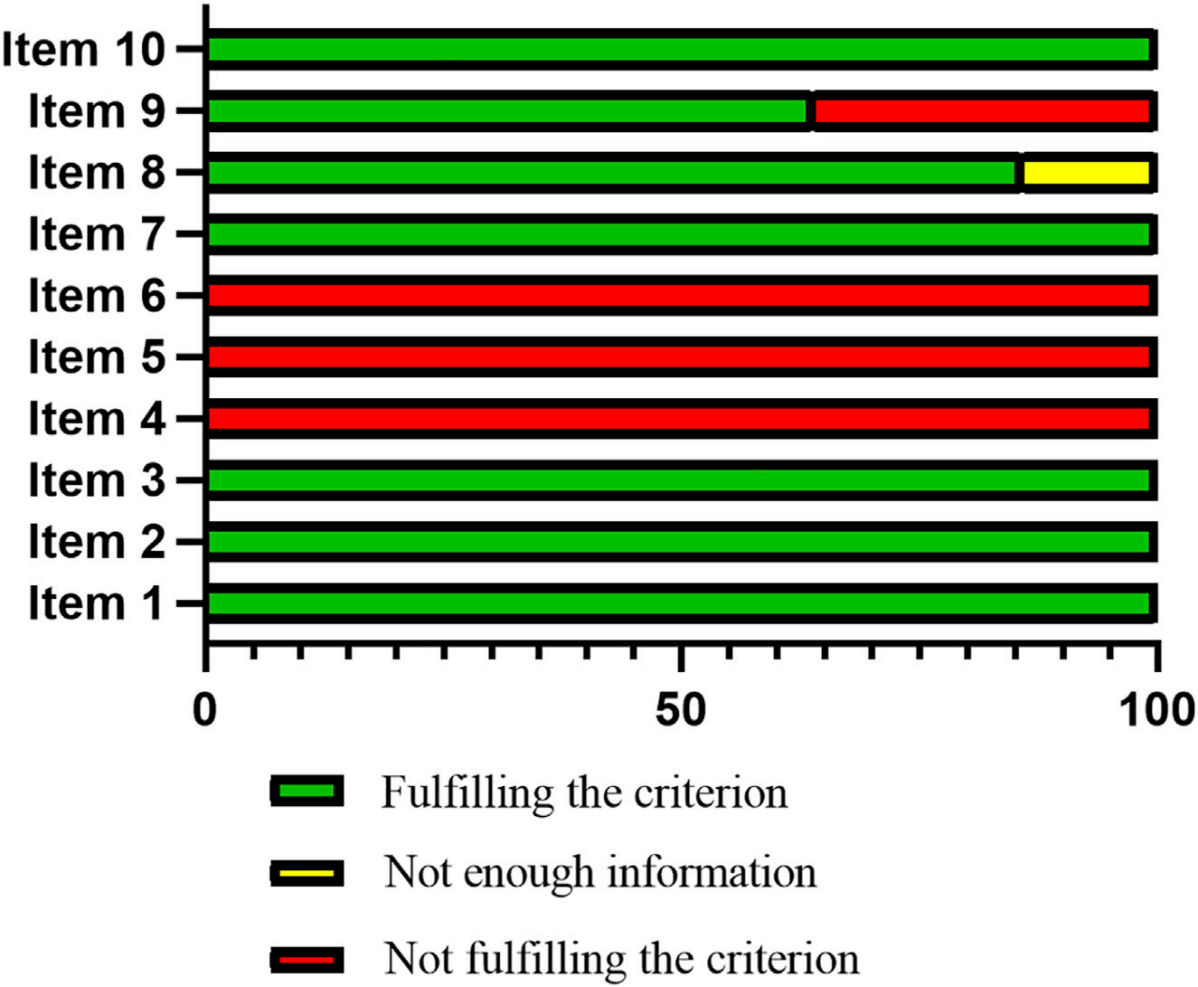
Schematic representation of the results of the risk of bias assessment.

Two tools were used to assess the quality of each study. Ten items were evaluated according to the CAMARADES checklist. With scores ranging from 5 to 7, the 14 included studies presented a quality score higher than 4, indicating that they were properly conducted. The 14 included studies are peer-reviewed publications and reported the presence of randomization of subjects into treatment groups, assessment of dose-response relationships, the statement of compliance with animal welfare regulations, and use of a suitable animal model. However, 2 studies (He Y. Y. et al., 2020; Fang et al., 2021) did not provide enough information to determine whether or not the avoidance of anaesthetic agents with marked intrinsic neuroprotective properties is fulfilled, and 5 studies (He Y. Y. et al., 2020; Liao et al., 2019; Mei et al., 2010; Fang et al., 2021; Mei et al., 2009) did not have the statements of potential conflict of interests. The median quality score indicated that 16 of the 21 secondary criteria had been reported fully. 3 studies (Liao, 2020; Liao et al., 2019; Xiang et al., 2024) did not provide the age of animals, and 2 studies (Mei et al., 2009; Ma et al., 2022) did not mention the sex of animals. The outcomes of the quality assessment are described in Tables 2, 3; Figure 2.

**TABLE 2:** The CAMARADES quality items.

Study	①	②	③	④	⑤	⑥	⑦	⑧	⑨	⑩	Quality score
He et al. (2020)	√	√	√	N	N	N	√	?	N	√	5
Liao (2020)	√	√	√	N	N	N	√	√	√	√	7
Liao et al. (2019)	√	√	√	N	N	N	√	√	N	√	6
Mei et al. (2010)	√	√	√	N	N	N	√	√	N	√	6
Wan (2022)	√	√	√	N	N	N	√	√	√	√	7
Sheraz (2019)	√	√	√	N	N	N	√	√	√	√	7
Fang et al. (2021)	√	√	√	N	N	N	√	?	N	√	5
Mei et al. (2009)	√	√	√	N	N	N	√	√	N	√	6
Ma et al. (2022)	√	√	√	N	N	N	√	√	√	√	7
Xiang et al. (2024)	√	√	√	N	N	N	√	√	√	√	7
He et al. (2020)	√	√	√	N	N	N	√	√	√	√	7
Ding et al. (2020)	√	√	√	N	N	N	√	√	√	√	7
Peng et al. (2022)	√	√	√	N	N	N	√	√	√	√	7
Liu et al. (2016)	√	√	√	N	N	N	√	√	√	√	7

(1) peer reviewed publication; (2) presence of randomization of subjects into treatment groups; (3) assessment of dose-response relationship; (4) blinded assessment of behavioural outcome; (5) monitoring of physiological parameters such as body temperature; (6) calculation of necessary sample size to achieve sufficient power; (7) statement of compliance with animal welfare regulations; (8) avoidance of anaesthetic agents with marked intrinsic neuroprotective properties (e.g., ketamine); (9) statement of potential conflict of interests; (10) use of a suitable animal model.

Abbreviations: √, fulfilling the criterion; N, not fulfilling the criterion; ?, not enough information to determine whether or not the criterion is fulfilled.

**TABLE 3:** Quality assessment of the included studies.

Study quality	He et al. (2020)	Liao (2020)	Liao et al. (2019)	Mei et al. (2010)	Wan (2022)	Sheraz (2019)	Fang et al. (2021)	Mei et al. (2009)	Ma et al. (2022)	Xiang et al. (2024)	He et al. (2020)	Ding et al. (2020)	Peng et al. (2022)	Liu et al. (2016)
Research question specified and clear?	√	√	√	√	√	√	√	√	√	√	√	√	√	√
Outcome measures relevant for AD research	√	√	√	√	√	√	√	√	√	√	√	√	√	√
Are the characteristics of study population clear?	N	N	N	N	N	N	N	N	N	N	N	N	N	N
Species	√	√	√	√	√	√	√	√	√	√	√	√	√	√
Background/generation	√	√	√	√	√	√	√	√	√	√	√	√	√	√
Sex (and distribution)	√	√	√	√	√	√	√	N	N	√	√	√	√	√
Age	√	N	N	√	√	√	√	√	√	N	√	√	√	√
Presence and correct control group?	√	√	√	√	√	√	√	√	√	√	√	√	√	√
Where the groups similar at baseline (if not randomized think of weight and sex, etc.)?	√	√	√	√	√	√	√	N	N	√	√	√	√	√
Is the experiment randomized?	√	√	√	√	√	√	√	√	√	√	√	√	√	√
Kind of supplement mentioned (tanshinone)?	√	√	√	√	√	√	√	√	√	√	√	√	√	√
Age when supplementation started mentioned?	√	N	N	√	√	√	√	√	√	N	√	√	√	√
Duration of supplementation clear and specified?	√	√	√	√	√	√	√	√	√	√	√	√	√	√
Amount of ginsenoside mentioned	√	√	√	√	√	√	√	√	√	√	√	√	√	√
Administration route specified	√	√	√	√	√	√	√	√	√	√	√	√	√	√
Is the timing of the supplementation during the day specified and similar in both groups?	√	√	√	√	√	√	√	√	√	√	√	√	√	√
Methods used for outcome assessment the same in both groups?	√	√	√	√	√	√	√	√	√	√	√	√	√	√
Did report animals who died or were otherwise removed from the study	N	N	N	N	N	N	N	N	N	N	N	N	N	N
Blinded outcome assessment?	N	N	N	N	N	N	N	N	N	N	N	N	N	N
Was the outcome assessment randomized across the groups?	N	N	N	N	N	N	N	N	N	N	N	N	N	N
Total number of animals included in statistical analyses clear?	√	√	√	√	√	√	√	√	√	√	√	√	√	√
Age of sacrificing animals mentioned?	√	√	√	√	√	√	√	√	√	√	√	√	√	√
Quality score (items√)	18	16	16	18	18	18	18	16	16	16	18	18	18	18

Abbreviations: √, fulfilling the criterion; N, not fulfilling the criterion; ?, not enough information to determine whether or not the criterion is fulfilled.

The methodology qualities of included studies were scores ranging from 5 to 7, indicating that some articles lacked rigorous animal experiment designs. Some methodological issues include: (1) Blinding: During surgical operations, the trial researchers were not blinded to the intervention that each animal received or to the physiological parameters being monitored. Double-blind research minimizes discrepancies in assessing the data and helps prevent the impact of the researcher’s subjective judgment on the findings. (2) Sample size: No study explained how to determine the sample size in order to verify that there was enough power. (3) Sequence generation: The precise sequence creation technique was not disclosed in any of the included investigations. In addition to preventing allocation bias, the creation of sequences in random assignment makes it easier to derive inferential inferences (Li et al., 2024).

### 3.4 Overall efficacy

As for each outcome, researchers considered all the experimental groups within each study instead of only a single representative group for each study, and the control group has been halved.

**FIGURE 3:**
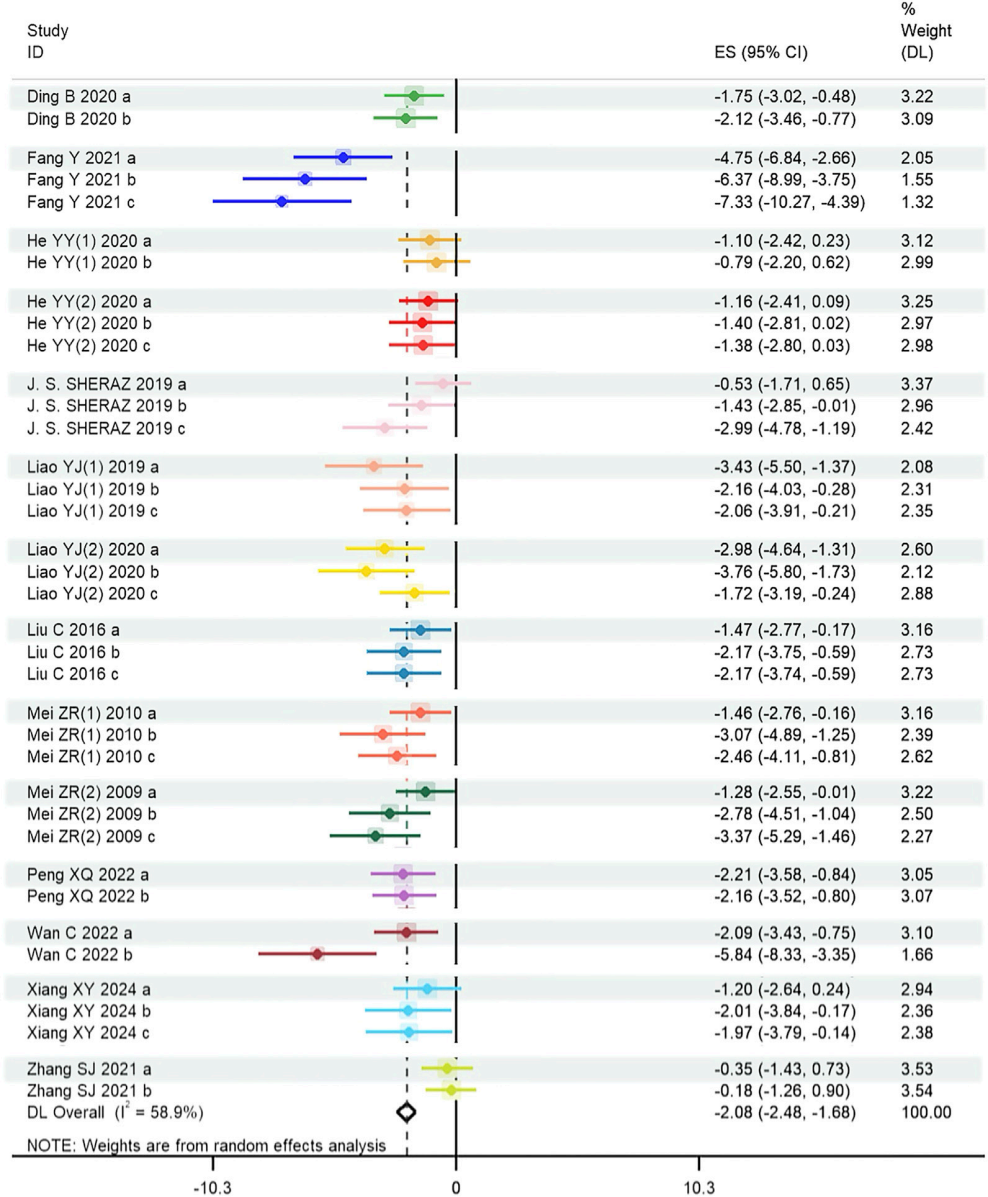
Effect of tanshinones treatment on escape latency (Heterogeneity: Tau^2^ = 0.825; Chi^2^ = 87.53, df = 36, P < 0.001; I^2^ = 58.87% and test for overall effect Z = 14.44, P < 0.001). Note: ES: Effect size; DL: DerSimonian-Laird method.

For reported escape latency, the results showed that tanshinones had a significantly beneficial effect on reduction of escape latency (SMD = −2.082, 95% CI = [−2.481, −1.683], p < 0.001), and the heterogeneity was significant (P < 0.001, I^2^ = 58.9%). Thus, a random effect model was applied for this analysis. The forest plot is shown in Figure 3.

**FIGURE 4:**
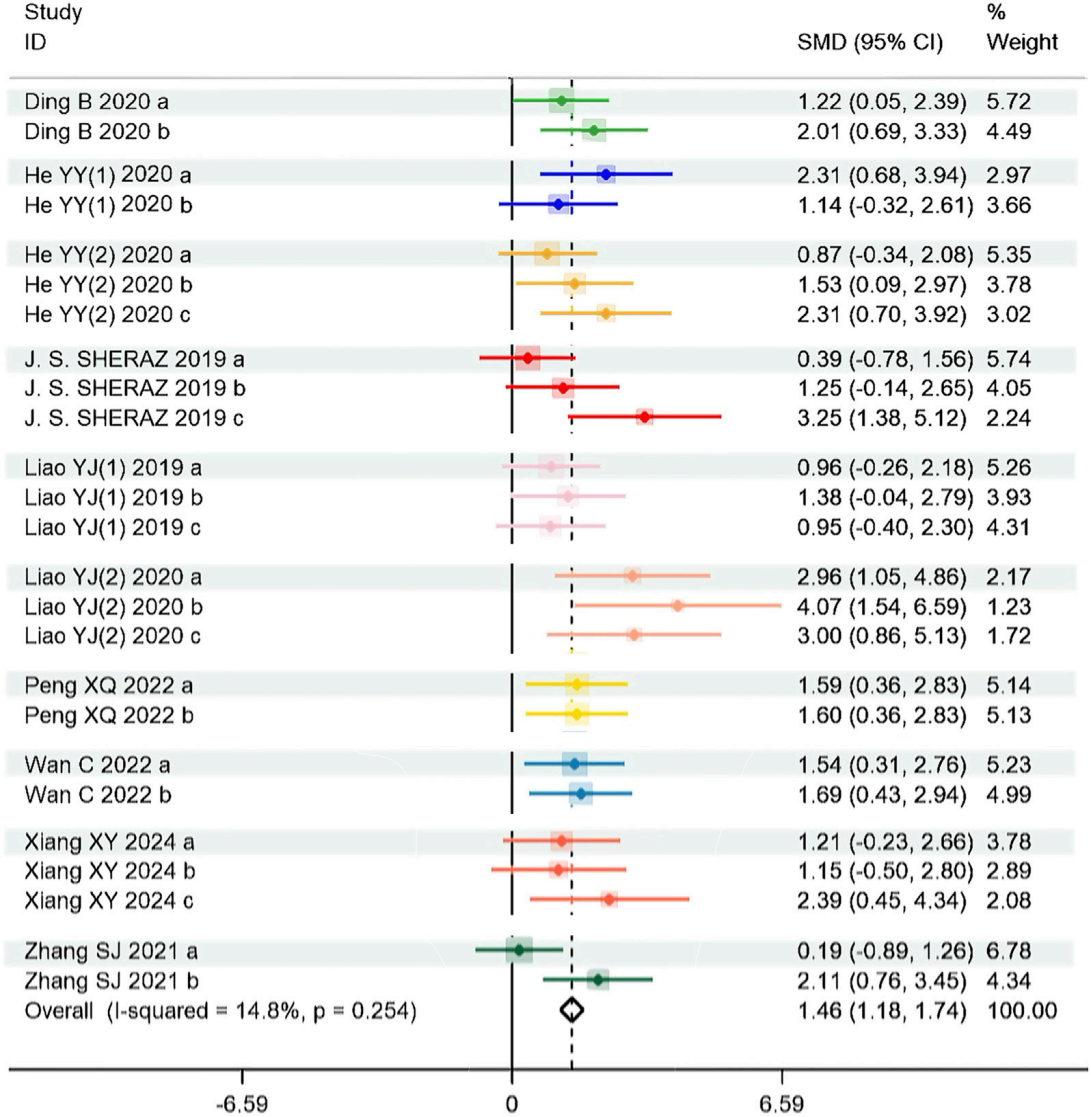
Effect of tanshinones treatment on platform crossing times (Heterogeneity: Chi^2^ = 28.16, df = 24, P = 0.254; I^2^ = 14.8% and test for overall effect Z = 10.24, P < 0.001). Note: SMD: Standard mean difference.

For platform crossing times, the results showed that tanshinones can increase the platform crossing times in AD animals [SMD = 1.464, 95% CI = (1.183, 1.744), p < 0.001]. A fixed effect model was applied for this analysis with low heterogeneity (P = 0.254, I^2^ = 14.8%). The forest plot is shown in Figure 4.

**FIGURE 5:**
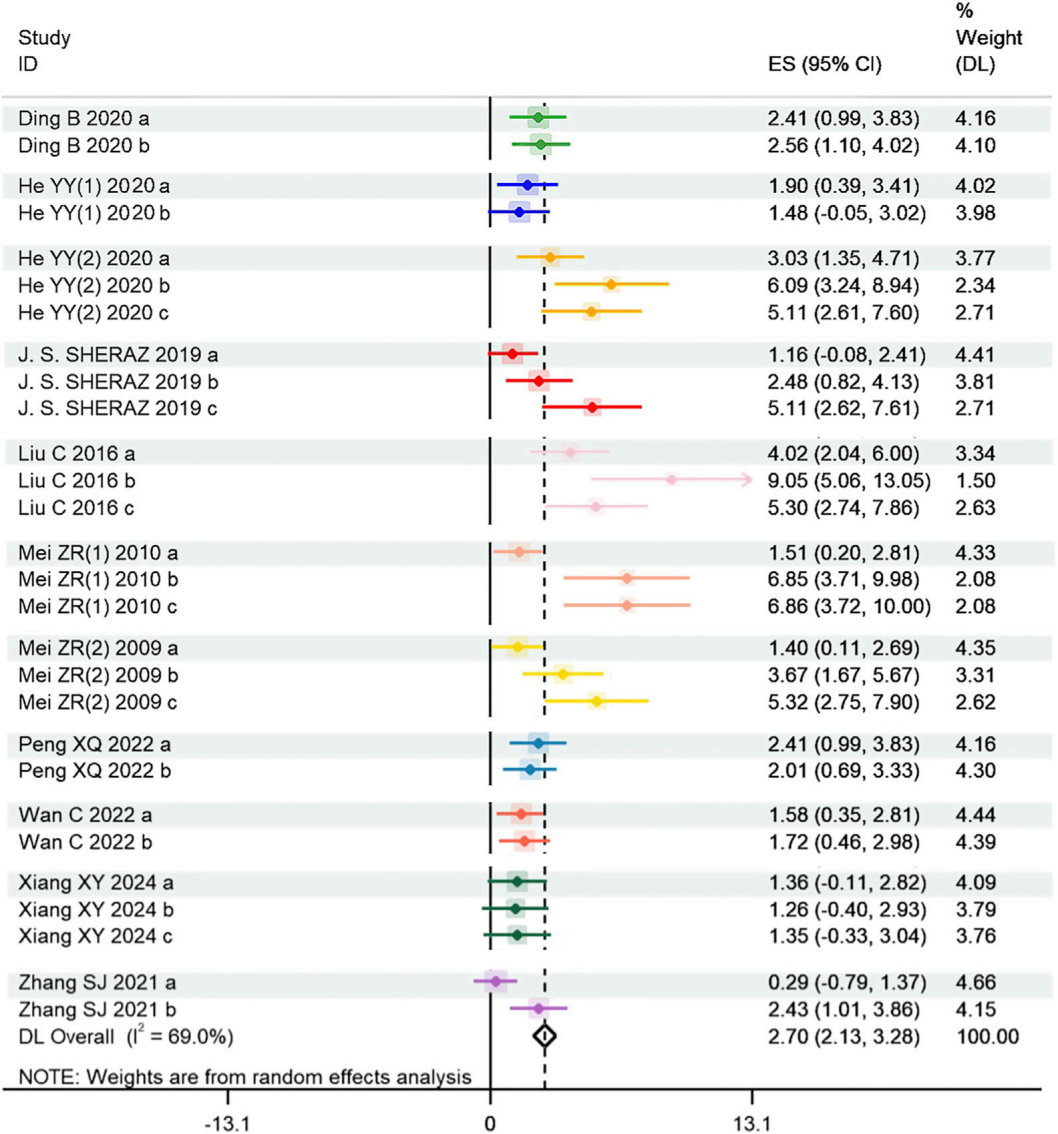
Effect of tanshinones treatment on time spent in target quadrants (Heterogeneity: Tau^2^ = 1.523; Chi^2^ = 87.15, df = 27, P < 0.001; I^2^ = 69.02% and test for overall effect Z = 14.15, P < 0.001). Note: ES: Effect size; DL: DerSimonian-Laird method.

For time in target quadrants, the global estimated effect of tanshinones was significantly beneficial [SMD = 2.703, 95% CI = (2.132, 3.275), p < 0.001], with significant heterogeneity among studies (P < 0.001, I^2^ = 69.0%). Thus, a random effect model was applied for this analysis. The forest plot is shown in Figure 5.

### 3.5 Subgroup analysis

Subgroup analysis was performed in order to determine the degree to which methodological differences between trials might have systematically influenced the variances in the principal treatment results. The stratifying characteristic is a substantial source of heterogeneity and could impact the treatment’s effectiveness, according to the general summary of each subgroup, which may then be analyzed for indicators of variance in the intervention’s effects (Amos et al., 2014). The findings of the subgroup analysis are described in Table 4.

**TABLE 4:** The results of subgroup analysis.

Subgroups	Escape latency			Platform crossing times			Time in target quadrants		
	Studies	SMD, 95%CI	p-Value	Studies	SMD, 95%CI	p-Value	Studies	SMD, 95%CI	p-Value
AD models									
Transgenic mice	3	−2.180 [−4.485.0.124]	<0.001	3	1.937 [1.312.2.561]	<0.001	3	2.354 [1.678.3.030]	<0.001
Non-transgenic mice and rats	6	−1.408 [−1.959, −0.858]	<0.001	5	1.083 [0.633.1.534]	<0.001	4	2.346 [0.978.3.714]	<0.001
Tanshinones									
Tanshinone IIA	8	−1.532 [−2.287, −0.778]	<0.001				8	2.309 [1.655.2.963]	<0.001
Cryptotanshinone	2	−2.210 [−3.019, −1.402]	<0.001				2	4.908 [2.738.7.077]	<0.001
Dosage									
dosage≤25 mg/kg	21	−2.201 [−2.830, −1.572]	<0.001	13	1.305 [0.949, 1.661]	<0.001	16	2.084 [1.543, 2.625]	<0.0001
25 mg/kg < dosage≤50 mg/kg	10	−2.002 [−2.522, −1.482]	<0.001	7	1.500 [0.936, 2.064]	<0.001	8	3.792 [2.229, 5.356]	<0.001
dosage>50 mg/kg	6	−1.974 [−2.639, −1.310]	<0.001	5	2.130 [1.364, 2.897]	<0.001	4	4.078 [1.868, 6.288]	<0.001
Route of administration									
Ip	11	−2.272 [−3.276, −1.267]	<0.001	8	1.408 [0.955, 1.861]	<0.001	8	1.867 [1.253, 2.480]	<0.001
Ig	23	−1.996 [−2.394, −1.598]	<0.001	17	1.498 [1.142, 1.855]	<0.001	17	3.006 [2.196, 3.815]	<0.001
Po	3	−2.155 [−3.092, −1.219]	<0.001				3	4.871 [0.737, 9.006]	0.021
Duration of the treatment									
duration <30 days	5	−1.591 [−2.095, −1.087]	<0.001	4	1.438 [0.646.2.230]	<0.001	3	2.831 [1.112.4.557]	<0.001
30 days ≤ duration <60 days	2	−1.225 [−2.597, 0.147]	0.001	2	1.060 [−0.279, 2.398]	0.005	2	1.858 [0.263, 3.738]	<0.001
duration ≥60 days	2	−2.401 [−6.950, 2.147]	0.009	2	2.026 [1.249, 2.802]	<0.001	2	2.202 [1.564, 3.057]	<0.001

#### 3.5.1 Animal model

In order to reduce the influence of dosage covariables on the analysis results, only one experimental group per each study with similar doses of 20 mg or 25 mg was included in this subgroup. Regarding the animal model used, significantly greater beneficial effects (p < 0.001) on acquisition memory and retention memory were observed in the transgenic mice (SMD = −2.180, SMD = 1.937, and SMD = 2.354). The forest plots can be viewed in Supplementary Material S2 1.1–1.3.

#### 3.5.2 Type of tanshinones

In order to reduce the influence of dosage covariables on the analysis results, only one experimental group per each study with similar doses of 15 mg or 20 mg was included in this subgroup. Concerning the protective effects of Tanshinone IIA and Cryptotanshinone administration on cognitive performance, a significant beneficial effect (p < 0.001) on acquisition memory was observed for both types of tanshinones (SMD = 1.532 and SMD = 2.210). Meanwhile, Cryptotanshinone treatment had a greater beneficial effect on retention memory (SMD = 4.908). The forest plots can be viewed in Supplementary Material S2 1.4–1.5.

#### 3.5.3 Dosage of tanshinones

An analysis was done on the effectiveness with which various tanshinone dosages affected cognitive function. All tanshinone dosages were found to have positive effects on both acquisition and retention memory. To ascertain whether the effects of smaller dosages are larger than those of higher dosages, the protective effects of 25 mg or lower doses on acquisition memory were investigated. On acquisition memory, a dosage of 25 mg or less was linked to a larger positive impact than a dosage above 25 mg (SMD = −2.201). In order to ascertain whether the effects of higher dosages are greater than those of smaller dosages, the protective effects of dosages over 50 mg on retention memory were also investigated, and a substantial effect was discovered. For retention memory, a dosage higher than 50 mg was linked to a noticeably higher positive result than a dose of less than 50 mg (SMD = 2.130 and SMD = 4.078). The forest plots can be viewed in Supplementary Material S2 1.6–1.8.

#### 3.5.4 Route of administration

Concerning the protective effects of route of administration on cognitive performance, although significant beneficial effects (p < 0.001) in the acquisition memory and retention memory were observed for all three administration routes, intragastric administration produced a smaller effect (SMD = −1.996) than either intraperitoneal or oral administration of tanshinones (SMD = −2.272 and SMD = −2.155). While both intraperitoneal and oral administration of tanshinones had a larger effect size for retention memory (SMD = 3.006 and SMD = 4.871). The forest plots can be viewed in Supplementary Material S2 1.9–1.11.

#### 3.5.5 Duration of the treatment

In order to reduce the influence of dosage covariables on the analysis results, only one experimental group per each study with similar doses of 20 mg or 25 mg was included in this subgroup. In relation to the duration of the treatment, the effect sizes for acquisition memory and retention memory were also investigated. For both acquisition and retention memory, there were notable variations in effect sizes between studies with a duration lower than 30 days and those with longer duration. Studies with a 60-day duration or longer had the largest effect size for acquisition memory (SMD = −2.401), and those with a 60-day duration or longer also had a larger effect size for retention memory (SMD = 2.026 and SMD = 2.202) than studies with a duration lower than 30 days. The forest plots can be viewed in Supplementary Material S2 1.12–1.14.

### 3.6 Meta-regression analysis

To further investigate the sources of heterogeneity within studies, meta-regression analyses were performed for escape latency, platform crossing times, and time in target quadrants results. For time in target quadrants, type of tanshinones, dosage of tanshinones, duration of treatment contributed to the heterogeneity (p = 0.030, p = 0.045, and p = 0.040). For platform crossing times, the AD animal model contributed to the heterogeneity (p = 0.021) However, the above factors did not have a significant impact on escape latency results. Meta-regression analysis figures can be viewed in Supplementary Material S2 2.1–2.14.

### 3.7 Publication bias

**FIGURE 6:**
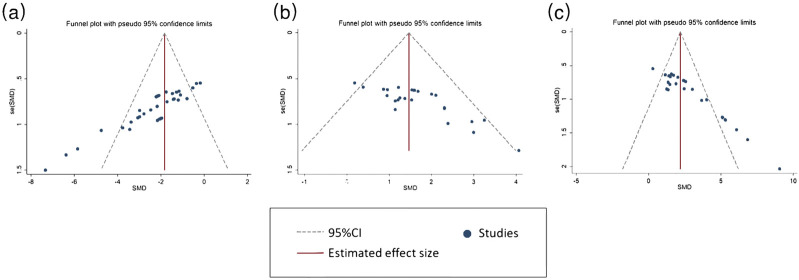
Funnel plot for escape latency **(A)**, platform crossing times **(B)** and time in target quadrants **(C)**. Note: SMD: Standard mean difference.

Lastly, small-study effects were identified, which could be a factor in publication bias. Sensitivity analysis and funnel plots were performed. Sensitivity analysis were performed to explore how the results would alter if one or more studies were excluded in the meta-analysis. The findings demonstrated that excluding one or more studies did not significantly alter the effects of tanshinones on acquisition memory and retention memory (Supplementary Material S2 3.1–3.3). Funnel plots showed an asymmetry for escape latency (Figure 6A), platform crossing times (Figure 6B) and time in target quadrants (Figure 6C) data, which illustrates possible negative results in the included studies were not reported (Egger regression, p < 0.001, p < 0.001 and p < 0.001, respectively). Considering the presence of significant publication bias, the trim and fill method was applied to verify the impact on results. The results suggest that the results of the meta-analysis are stable [SMD = −1.965, 95% CI = (−2.340, −1.590), p < 0.001; SMD = 1.286, 95% CI = (0.943, 1.623), p < 0.001; and SMD = 2.466, 95% CI = (1.780, 3.152), p < 0.001] and that publication bias has little effect on the results of this study.

### 3.8 An overview of the proposed mechanism of tanshinones in animal models of AD

Every study that was included offered comprehensive details regarding potential tanshinones therapy mechanisms in animal models of AD. All of the suggested mechanisms are included in Table 5.

**TABLE 5:** Proposed mechanism of tanshinones.

Study	Proposed mechanisms
He et al. (2020)	Reducing expression of CHOP, phosphorylated JNK, Bax and cleaved Caspase-3 in cortex and hippocampus. Effectively inhibited ERS and its cellular apoptosis
Liao (2020)	Reducd IL-1β and IL-6 inflammatory factors in the hippocampus. Moreover, reduced apoptosis and protected hippocampal nerve cells by regulating apoptosis factors Caspase-3,Caspase-8,and Caspase-9
Liao et al. (2019)	Increased SOD and GSH-Px activities and decreased MDA levels, and downregulated Caspase-3, Caspase-8 and Caspase-9 mRNA levels
Mei et al. (2010)	Scavenged MDA, a metabolite of lipid peroxidation in the body, and enhancing the activities of SOD and GSH-Px, which improves the antioxidant capacity of the body
Wan (2022)	Decreased MDA levels, and downregulated Caspase-3, Caspase-8 and Caspase-9 levels to relieve oxidative stress, inhibit neurodegeneration, and attenuate neuronal apoptosis
Sheraz (2019)	Inhibited the phosphorylation of Y216-GSK-3β and promoted the phosphorylation of S9-GSK-3β
Fang et al. (2021)	Activated of the PI3K/AKT pathway and a reduction in brain inflammation, which would inhibit the excessive activation of microglia and astrocytes
Mei et al. (2009)	Upregulated α-secretase protein expression, increased the release of sAPPɑ and decreased Aβ levels through the PI3K/AKT pathway
Ma et al. (2022)	Reduced the levels of ROS and MDA, while improved the activity of SOD in both hippocampus and cortex. In addition, inhibited the activity of AChE, while improved the activity of ChAT. Elevated the protein expressions of neurotrophic factors (BDNF and NGF) and synapse-related proteins (PSD93, PSD95 and SYP) in both the hippocampus and cortex
Xiang et al. (2024)	Upregulate the expression of the CREB-BDNF-TrkB signaling pathway in the hippocampus of brain tissue, produce anti-neuro inflammatory, antioxidant stress, inhibit neuronal apoptosis effects, and improve cholinergic neurotransmitter disorder
He et al. (2020)	Prevented abnormal expression of glucose regulated protein 78, initiation factor 2α, inositol-requiring enzyme 1α, activating transcription factor 6, as well as suppressed the activation of CHOP and JNK pathways in the parietal cortex and hippocampus
Ding et al. (2020)	Attenuated Aβ accumulation, synapse-associated proteins and neuronal loss, as well as periplaque microgliosis and astrocytosis in the cortex and hippocampus. Furthermore, tan IIA significantly suppressed NF-κB signaling pathway and the production of pro-inflammatory cytokines (TNF-α, IL-6, and IL-1β)
Peng et al. (2022)	Attenuated tau hyperphosphorylation and prevented neuronal loss and apoptosis in the parietal cortex and hippocampus, as well as reversed cholinergic dysfunction and reduced oxidative stress
Liu et al. (2016)	Reduced STZ induced elevation in AChE activity and malondialdehyde MDA level, and significantly inhibited STZ induced reduction in SOD and glutathione peroxidase GSH-Px activities in the parietal cortex and hippocampus

Abbreviations: CHOP, C/EBP homologous protein; JNK, c-JunN-terminal kinase; ERS, endoplasmis reticulum stress; SOD, superoxide dismutase; GSH-Px, glutathione peroxidase; MDA, malondialdehyde; sAPPα, a secreted fragment of APP; Aβ, β-amyloid; ROS, reactive oxygen species; AChE, acetylcholinesterase; ChAT, choline acetyltransferase; BDNF, anti-brain-derived neurotrophic factor; NGF, anti-nerve growth factor; PSD95, anti-postsynaptic density 95; PSD93, anti-postsynaptic density 93; SYP, anti-synaptophysin; CREB, cyclic adenosine monophosphate response element binding protein; TrkB, tyrosine kinase receptor protein; NF-κB, nuclear factor-κB; TNF-α, tumor necrosis factor-α; IL-6, interleukin-6; IL-1β, interleukin-1β.

## 4 Discussion

### 4.1 Summary of findings

For thousands of years, Chinese people have been treated with herbal formulae. Currently organized alongside the traditional biomedical approaches of western medicine, traditional Chinese medicine is an essential component of the Chinese mainstream healthcare system. In traditional Chinese medicine theory, tanshinones effectively improve the stagnation of qi and blood in the patient’s body and restore the function of internal organs and meridians (Pharmacopoeia of the People’s Republic of China, 2020). But there is still not yet adequate evidence of tanshinones as a treatment program for AD. In order to assess the effectiveness of tanshinones treatment for AD and to comprehend the underlying mechanisms, we included 14 animal investigations. Tanshinones have neuroprotective properties that improve cognitive outcomes in AD, according to the findings of this systematic review and meta-analysis.

Our analysis further indicates that the design of the original research has an influence on the research results. Primarily, the effect values of transgenic mice were higher than those of non-transgenic mice and rats, indicating that animals of different modeling methods may have different responses to tanshinones. The possible reasons are as follows: Transgenic mice might be genetically engineered to express certain genes that mimic human diseases or conditions, such as AD. In the case of tanshinones, which are bioactive compounds derived from the Salvia miltiorrhiza plant, their efficacy may be heightened in transgenic models because these mice exhibit disease-specific pathways and biomarkers that are more similar to human conditions. This enhanced relevance of disease models makes transgenic mice more sensitive to treatments like tanshinones, which may have targeted effects on the molecular pathways affected by diseases like Alzheimer’s (He Y. et al., 2020). In contrast, non-transgenic mice or rats do not carry the genetic alterations associated with such diseases, which might lead to less pronounced effects when treated with tanshinones. Without these disease-specific markers, the compounds might not engage with the molecular pathways in the same way, thereby resulting in weaker or less significant effects (Li et al., 2016). Another factor that might contribute to the difference in effectiveness between transgenic and non-transgenic mice and rats is the pharmacokinetics of tanshinones. The absorption, distribution, metabolism, and excretion of a compound like tanshinone can vary between different species and even between genetically modified and non-modified animals. Transgenic mice might metabolize the compound in a way that enhances its bioavailability or its ability to reach the target sites in the brain. Non-transgenic animals, on the other hand, might not exhibit the same level of absorption or target site engagement, potentially leading to a lower therapeutic effect (Song et al., 2023).

Compared to Tanshinone IIA treatment, Cryptotanshinone treatment enhanced memory retention. While most of the studies used Tanshinone IIA, the effect values of Cryptotanshinone were probably affected by the experimental design since the studies were conducted by the same author. The lack of observation of the significance of the effect of Cryptotanshinone in retention memory probably resulted from the reduced number of studies using this treatment. It would be desirable to increase the number of studies using the Cryptotanshinone treatment; however, no more studies using this model were found that met the inclusion criteria.

Considering the dosage of tanshinones also affects the therapeutic effect. A 25 mg or lower dosage was associated with a greater beneficial effect on acquisition memory, while a dosage higher than 50 mg had a greater beneficial outcome for retention memory. The differential effects of tanshinones at varying dosages may be attributed to several pharmacological mechanisms. Lower dosages might facilitate the balance of neurotransmitters, such as acetylcholine, which is crucial for memory formation and retrieval. Higher doses, conversely, may lead to an oversaturation of these pathways, impeding optimal neurotransmitter function (Liao et al., 2020). Moreover, low doses of tanshinones can exhibit anti-inflammatory properties, which are essential for protecting neural tissues from the chronic inflammation typical in Alzheimer’s pathology. Higher doses, however, can trigger compensatory mechanisms that might exacerbate inflammation (Song et al., 2023).

Considering the administration route of tanshinones, intragastric administration produced a smaller effect than either intraperitoneal or oral administration of tanshinones. The possible reasons are as follows: The ability of tanshinones to reach the brain is crucial for their effects on cognitive performance, particularly in Alzheimer’s disease models where neurodegeneration and cognitive decline are key features. The blood-brain barrier (BBB) is a selective permeability barrier that controls the entry of substances into the brain. Certain administration routes, such as intraperitoneal and oral administration, may lead to a more efficient transport of tanshinones across the BBB, especially if the compounds are small, lipophilic, or have specific transport mechanisms that facilitate their passage. Intraperitoneal administration allows tanshinones to enter the bloodstream more rapidly and distribute throughout the body. The fast distribution increases the likelihood of tanshinones reaching the brain in higher concentrations, where they can exert their effects on neuronal function and cognitive processes. Although oral administration does involve absorption via the gastrointestinal tract, certain formulations can enhance the bioavailability and brain penetration of compounds. For instance, oral formulations like nanoparticles, liposomes, or modified-release systems can improve the absorption of tanshinones and increase their bioavailability, enhancing their effects on the brain. By contrast, intragastric administration may result in slower and less efficient absorption, meaning lower concentrations of tanshinones in the bloodstream and consequently, lower concentrations reaching the brain. This can result in diminished effects on cognitive performance (Zhang, 2019).

Studies with a 60-day duration or longer demonstrated a stronger protective impact on both acquisition and retention memory, according to our systematic review. The possible reasons are as follows: Long-term tanshinone treatment may increase the production of neurotrophic factors such as brain-derived neurotrophic factor (BDNF). These factors play a pivotal role in promoting neuronal survival, growth, and synaptic function. Sustained elevation of BDNF levels could enhance memory acquisition and retention over time. Another factor is synaptic plasticity: Prolonged exposure to tanshinones could facilitate enhanced synaptic plasticity, which is essential for memory processes. Long-term treatment may lead to structural changes in the brain, such as increased dendritic branching and synapse formation, both of which contribute to improved memory acquisition and retention in AD models (Meier et al., 2020).

Given the limited alternatives available for treating AD, the results of this meta-analysis are interesting since they demonstrate the considerable anti-cognitive impairment impact of tanshinones against AD in animal models, raising the possibility that treating AD patients with tanshinones may improve their cognitive impairment and emphasizing the need for more research, including human clinical trials.

### 4.2 Possible drug protection mechanism analysis

#### 4.2.1 Regulation of APP metabolism and Aβ deposition

Cryptotanshinone was found to upregulate α-secretase activity, which promotes the non-amyloidogenic processing of amyloid precursor protein (APP). This leads to increased release of the neuroprotective secreted fragment of APP (sAPP) and decreased generation of Aβ. In APP/PS1 transgenic mice, Cryptotanshinone treatment reduced Aβ plaque deposition in the brain and improved spatial learning and memory abilities (Mei et al., 2009; Mei et al., 2010).

#### 4.2.2 Inhibition of neuroinflammation

Tanshinone IIA was shown to suppress neuroinflammation, which plays a crucial role in AD pathogenesis. It inhibited the activation of the receptor for advanced glycation end products (RAGE)/nuclear factor-κB (NF-κB) signaling pathway. In APP/PS1 mice and cultured BV2 and U87 cells, Tanshinone IIA treatment reduced the number of activated microglia and astrocytes, as well as the production of pro-inflammatory cytokines such as TNF-α, IL-6, and IL-1β (Ding et al., 2020; Peng et al., 2022).

#### 4.2.3 Alleviation of oxidative stress

Tanshinone IIA and Cryptotanshinone exhibited antioxidant effects in AD mice. They increased the activities of antioxidant enzymes such as superoxide dismutase (SOD) and glutathione peroxidase (GSH-Px) and decreased the level of malondialdehyde (MDA). In APP/PS1 transgenic mice models with intracerebroventricular streptozotocin-induced memory deficits, treatment with these compounds reversed the oxidative stress-induced changes in the brain (Liu et al., 2016).

#### 4.2.4 Modulation of tau phosphorylation

Tanshinone IIA was found to regulate the glycogen synthase kinase-3β (GSK-3β)-related signaling pathway. It activated the phosphatidylinositol 3-kinase/protein kinase B (PI3K/Akt) signaling pathway, which in turn inhibited GSK-3β activity. This led to decreased tau hyperphosphorylation in the parietal cortex and hippocampus of APP/PS1 mice (Peng et al., 2022).

#### 4.2.5 Improvement of cholinergic function

Tanshinone IIA inhibited neuronal apoptosis in the brain of AD mice. It regulated the expression of apoptotic genes and proteins, such as increasing the ratio of Bcl-2/Bax and reducing the level of cleaved caspase-3. In APP/PS1 transgenic mice, Tanshinone IIA treatment protected neurons from apoptosis and prevented neuronal loss (He Y. et al., 2020; He Y. Y. et al., 2020).

#### 4.2.6 Enhancement of synaptic plasticity

Tanshinone IIA increased the expressions of synapse-associated proteins such as synaptophysin (Syn) and postsynaptic density 95 (PSD95) in the brains of AD mice. This suggests that it may enhance synaptic plasticity and improve cognitive function. In APP/PS1 mice, Tanshinone IIA treatment reversed the decrease in Syn and PSD95 levels, indicating its beneficial effect on synaptic function (Ma et al. (2022)).

#### 4.2.7 Regulation of endoplasmic reticulum stress

Tanshinone IIA was shown to alleviate endoplasmic reticulum (ER) stress in AD mice. It reduced the expression of ER stress markers such as glucose-regulated protein 78 (GRP78) and inhibited the activation of the unfolded protein response (UPR) signaling pathways. In APP/PS1 transgenic mice and SH-SY5Y cells, Tanshinone IIA treatment inhibited ER stress-induced apoptosis (He Y. et al., 2020; He Y. Y. et al., 2020).

### 4.3 Limitations

The following were some of the limitations of the system review: (1) The meta-analysis’s limited selection of literature may have influenced its findings, which may have a certain bias in the conclusion. (2) We only assessed how tanshinones affected AD animals’ cognitive deficits. Due to a lack of data, we did not perform analyses specifically aimed at the impact on histology, such as plaques and tangles. (3) Due to language barriers, we failed to search for studies published in other languages, such as Japanese, and instead only searched databases for publications published in Chinese or English. We might have overlooked any pertinent papers because Japan is one of the major Danshen nations. (4) The literature lacked descriptions of experimental animals’ adverse reactions.

The number of preclinical experiments conducted annually continues to rise, and our understanding of the disease mechanism is improving. However, because of the translational paradigm’s shortcomings, the number of innovative treatments for AD that make it to the clinic keeps declining (Sena et al., 2014). Experts from a variety of scientific disciplines advocate the standardization of animal protocols and the systematic review of animal models that do not currently qualify as predictive modalities for human responses to drugs and disease (Greek and Menache, 2013). Therefore, these limited results may not be sufficient for the transition from animal experiments to human clinical trials. Consequently, high methodological reporting and quality control experimental trials are required to properly assess the impact of this intriguing pharmaceutical intervention before any clinical practice recommendations are performed.

## 5 Conclusion

This systematic review and meta-analysis indicate that tanshinones therapy can alleviate cognitive impairment in experimental animal models of AD. Tanshinones may have a neuroprotective effect in AD, despite the fact that certain aspects, such as the quality of the study and possible publication bias, may raise questions about the validity of these positive results. However, when evaluated in extensive, expensive, and time-consuming human clinical trials, innovative neuroprotective medications may turn out to be unsuccessful in the absence of rigorous, robust, and thorough preclinical studies. Thus, further thoughtfully designed and well-reported experimental animal research is required.

## Data Availability

The original contributions presented in the study are included in the article/Supplementary Material, further inquiries can be directed to the corresponding author.
