# Supplementary material for: The effect of tanshinones on cognitive impairments in animal models of Alzheimer’s disease: a systematic review and meta-analysis
Source: Front Pharmacol. 2025 Feb 27;16:1529327. doi: 10.3389/fphar.2025.1529327 (PMC11904413; doi:10.3389/fphar.2025.1529327)
Supplement: Supplementary file 1 [file DataSheet1.pdf]

Search Strategy

PubMed:
(("Salvia miltiorrhiza"[Mesh]) OR ("tanshinone" [Title/Abstract])) AND (((((Alzheimer's[Title/Abstract]) OR (Alzheimer[Title/Abstract])) OR (dementia[Title/Abstract])) OR (cognition[Title/Abstract])) OR (cognitive[Title/Abstract]))

China National Knowledge Infrastructure：
主题:(痴呆 or 阿尔茨海默症 or 阿尔茨海默病 or 认知障碍) and 主题：（丹参酮）

Web of science:
1: (TS=(Salvia miltiorrhiza)) OR TS=(tanshinone)
2: ((((TS=(Alzheimer's)) OR TS=(Alzheimer)) OR TS=(Cognition)) OR TS=(cognitive))
3:#1 AND #2

EMBASE:
#1. 'dementia':ab,ti OR 'alzheimer':ab,ti OR 'cognition':ab,ti OR 'cognitive':ab,ti
#2. 'tanshinone':ab,ti OR 'salvia miltiorrhiza':ab,ti
#3. #1 AND #2

Chinese Biomedicine Database:
序号 检索表达式 命中文献数 检索时间
1) "阿尔茨海默症"[常用字段:智能] OR "阿尔茨海默病"[常用字段:智能] OR "痴呆"[常用字段:智能] OR "认知障碍"[常用字段:智能]
2) "丹参"[常用字段:智能] OR "丹参酮"[常用字段:智能]
3) (#2) AND (#1)

Cochrane Library:
#1 (Dementia):ti,ab,kw OR (Alzheimer's):ti,ab,kw OR (Alzheimer):ti,ab,kw OR (Cognition):ti,ab,kw OR (cognitive):ti,ab,kw
#2 (tanshinone):ti,ab,kw OR (Salvia miltiorrhiza):ti,ab,kw
#3 #1 and #2
