# Supplementary material for: The effect of tanshinones on cognitive impairments in animal models of Alzheimer’s disease: a systematic review and meta-analysis
Source: Front Pharmacol. 2025 Feb 27;16:1529327. doi: 10.3389/fphar.2025.1529327 (PMC11904413; doi:10.3389/fphar.2025.1529327)

Supporting information for data analysis

## 1 Subgroup analysis forest plots

## Subgroup analysis of escape latency after doses of 20 mg or 25 mg of **tanshinones** intervention according to AD model


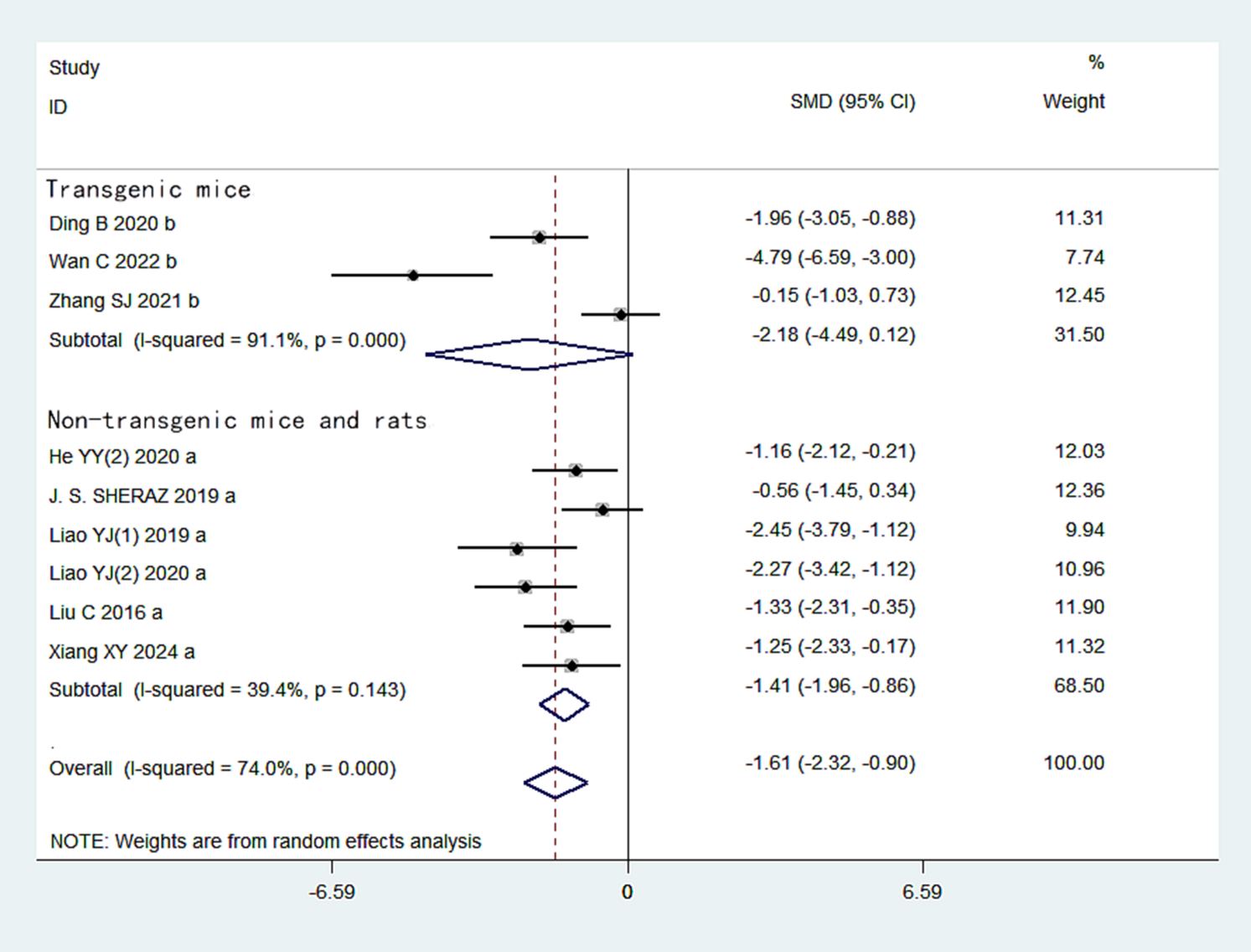


## Subgroup analysis of platform crossing times after doses of 20 mg or 25 mg of **tanshinones** intervention according to AD model


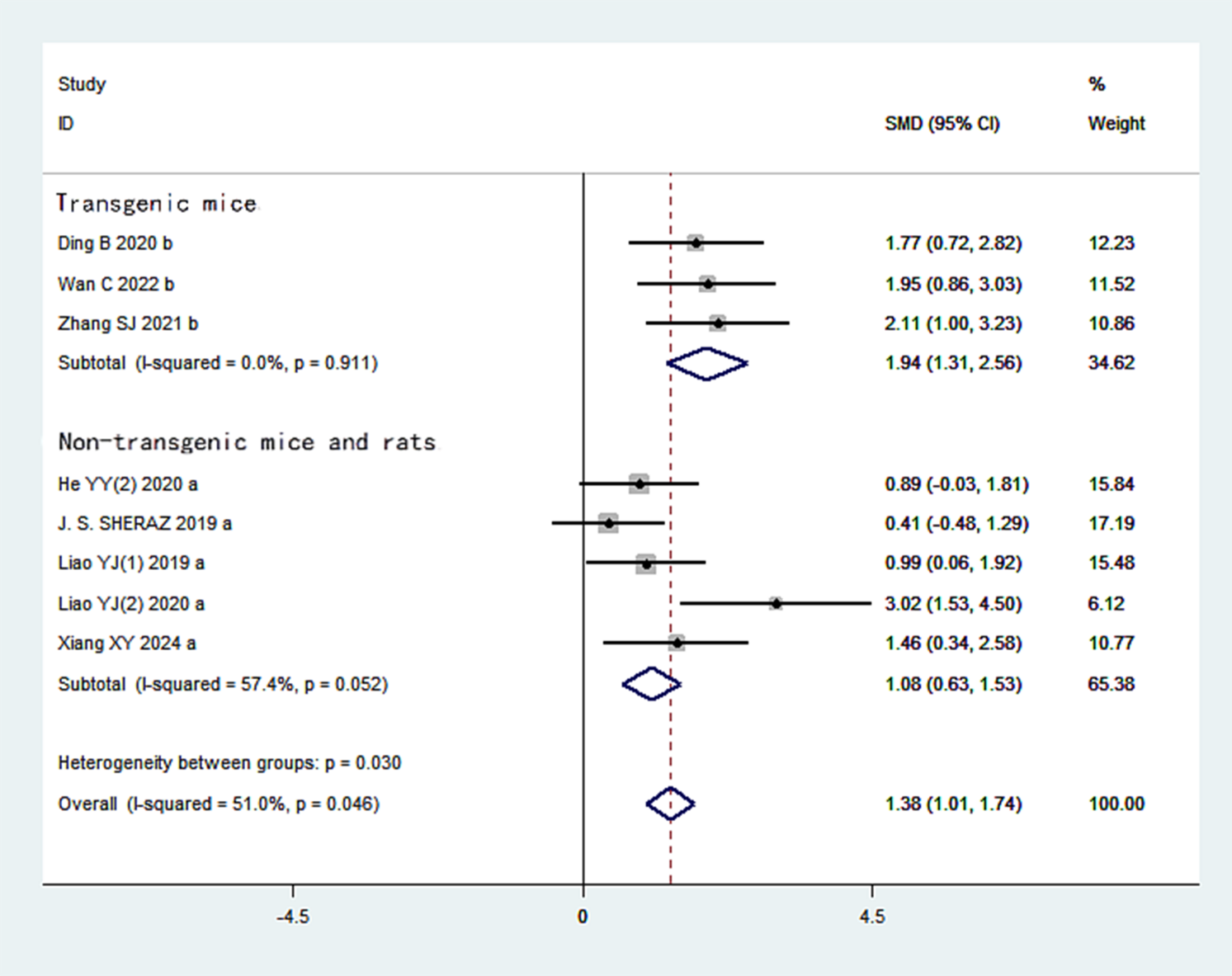


## Subgroup analysis of time in target quadrants after doses of 20 mg or 25 mg of **tanshinones** intervention according to AD model

##
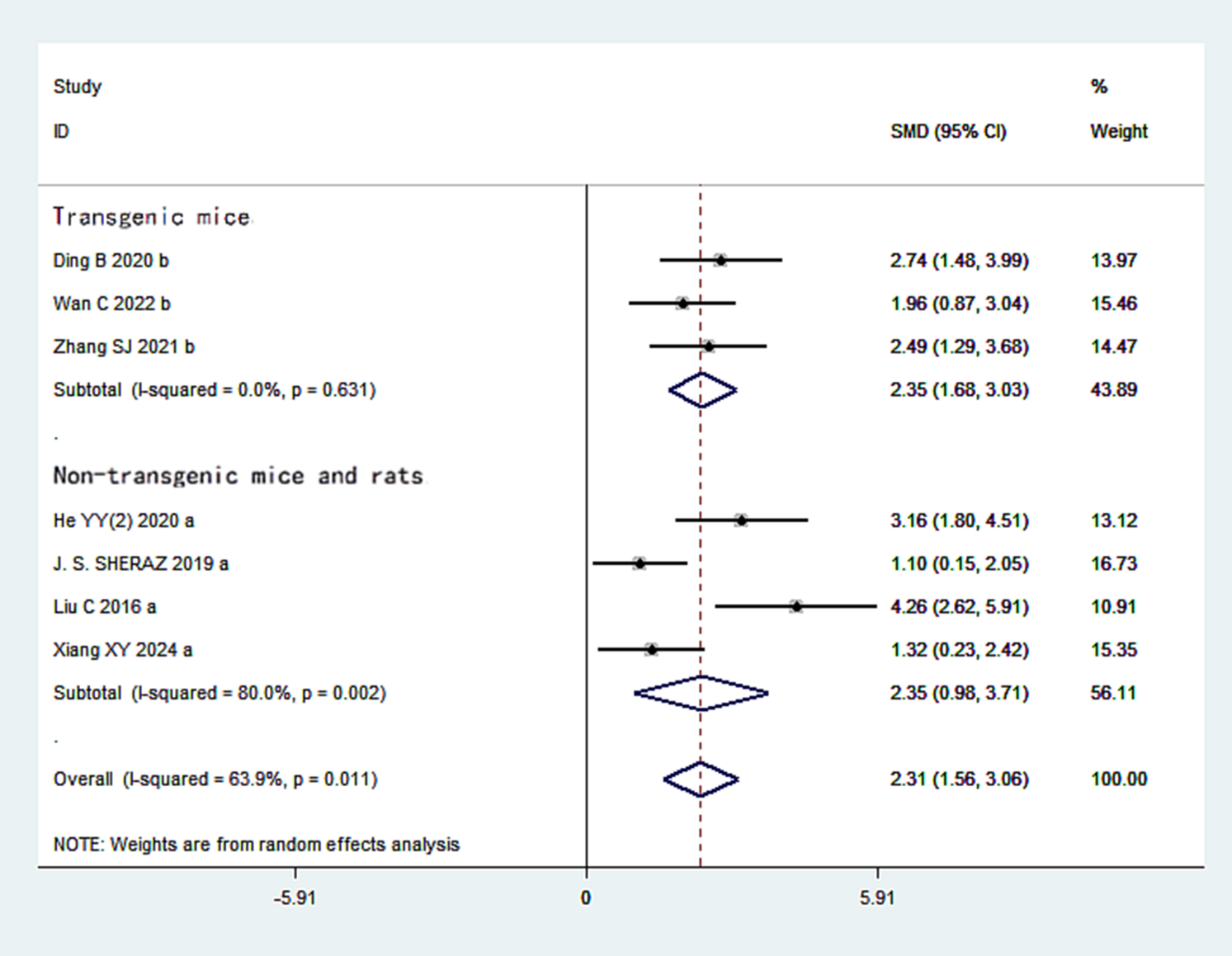


## Subgroup analysis of escape latency after doses of 15 mg or 20 mg of **tanshinones** intervention according to **the type of tanshinones**


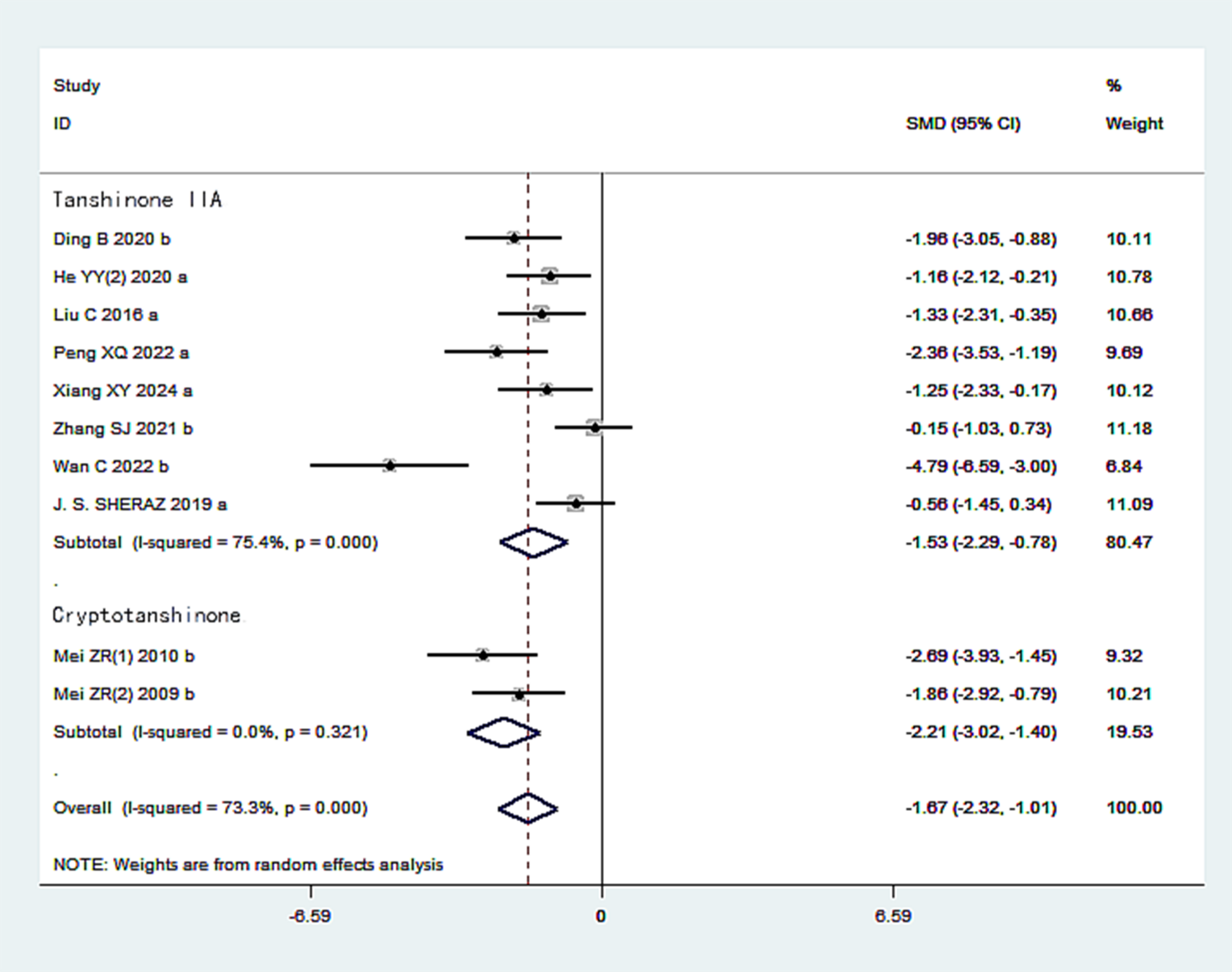


## Subgroup analysis of time in target quadrants after doses of 15 mg or 20 mg of **tanshinones** intervention according to **the type of tanshinones**


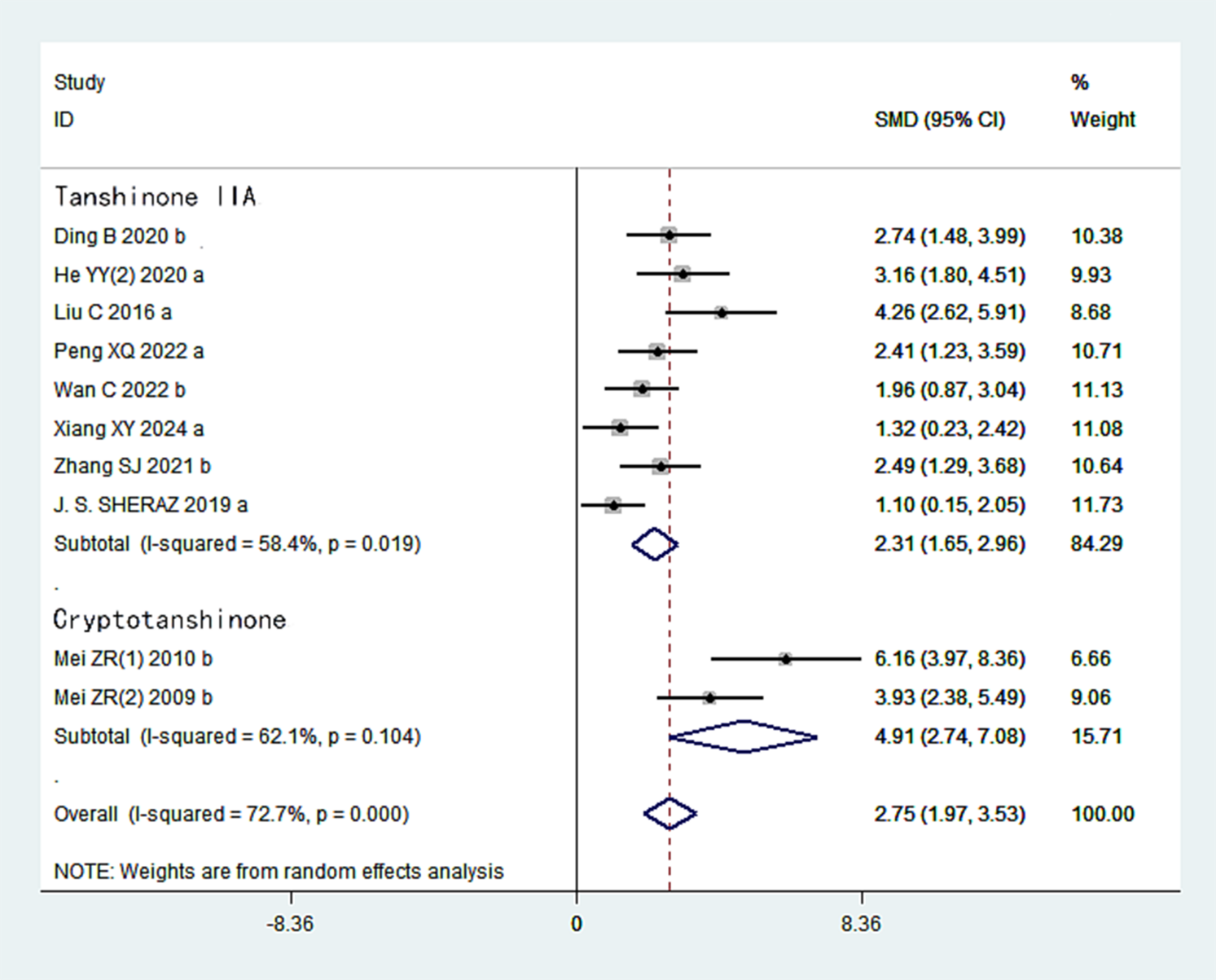


## Subgroup analysis of escape latency after **tanshinones** intervention according to **the dosage of tanshinones**


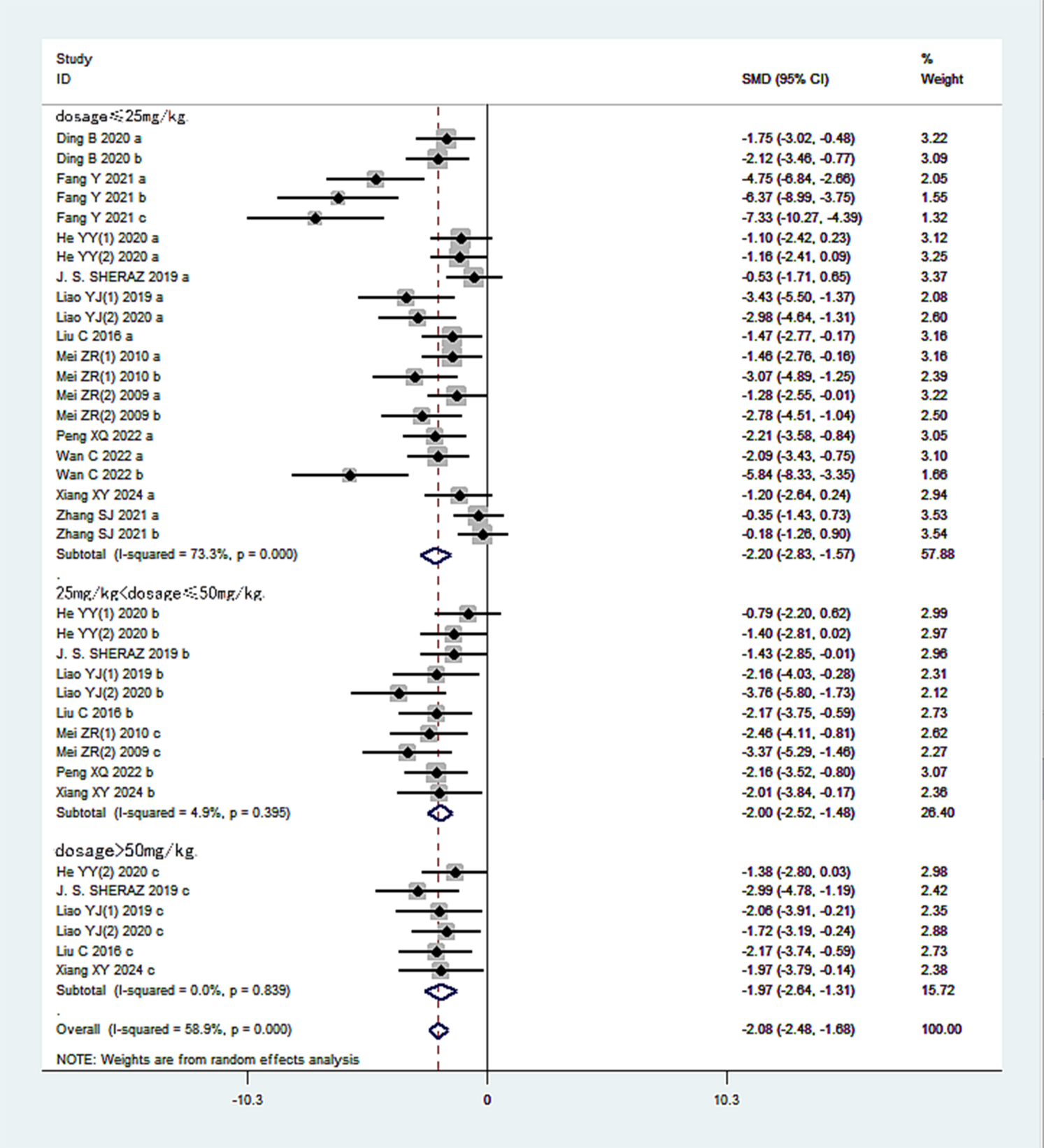


## Subgroup analysis of platform crossing times after **tanshinones** intervention according to **the dosage of tanshinones**

##
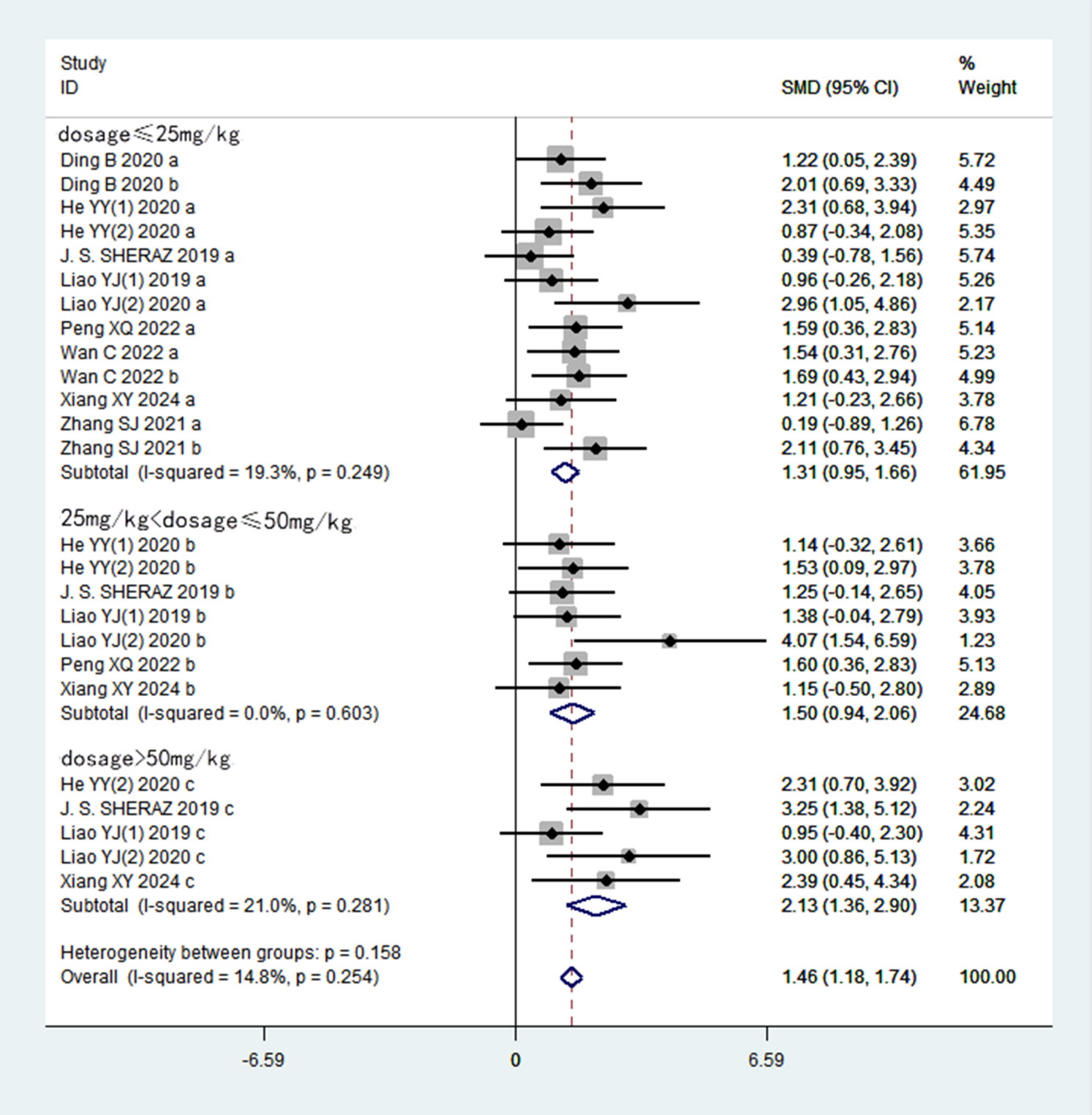


## Subgroup analysis of time in target quadrants after **tanshinones** intervention according to **the dosage of tanshinones**


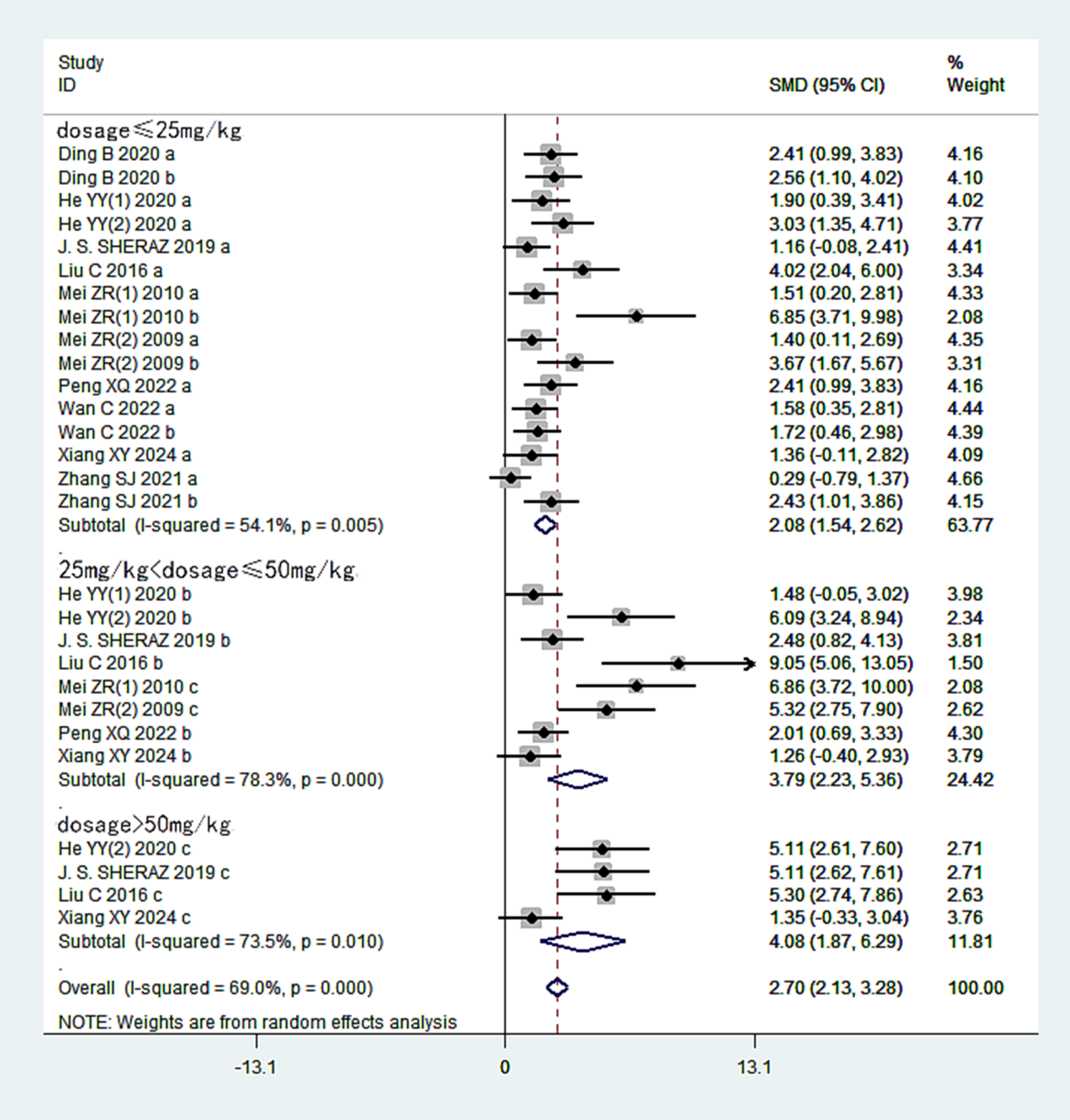


## Subgroup analysis of escape latency after **tanshinones** intervention according to **the route of administration**


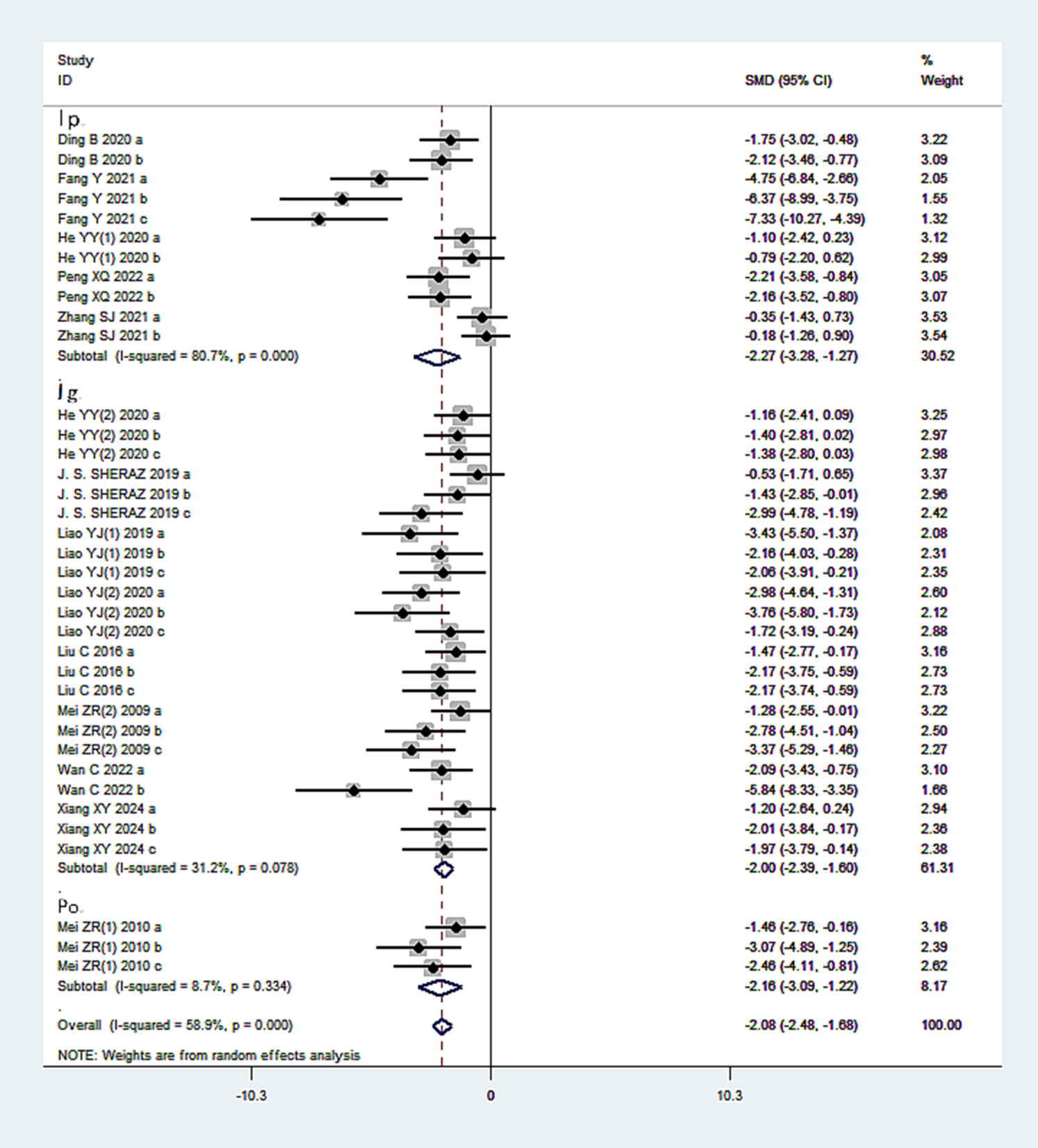


## Subgroup analysis of platform crossing times after **tanshinones** intervention according to **the route of administration**


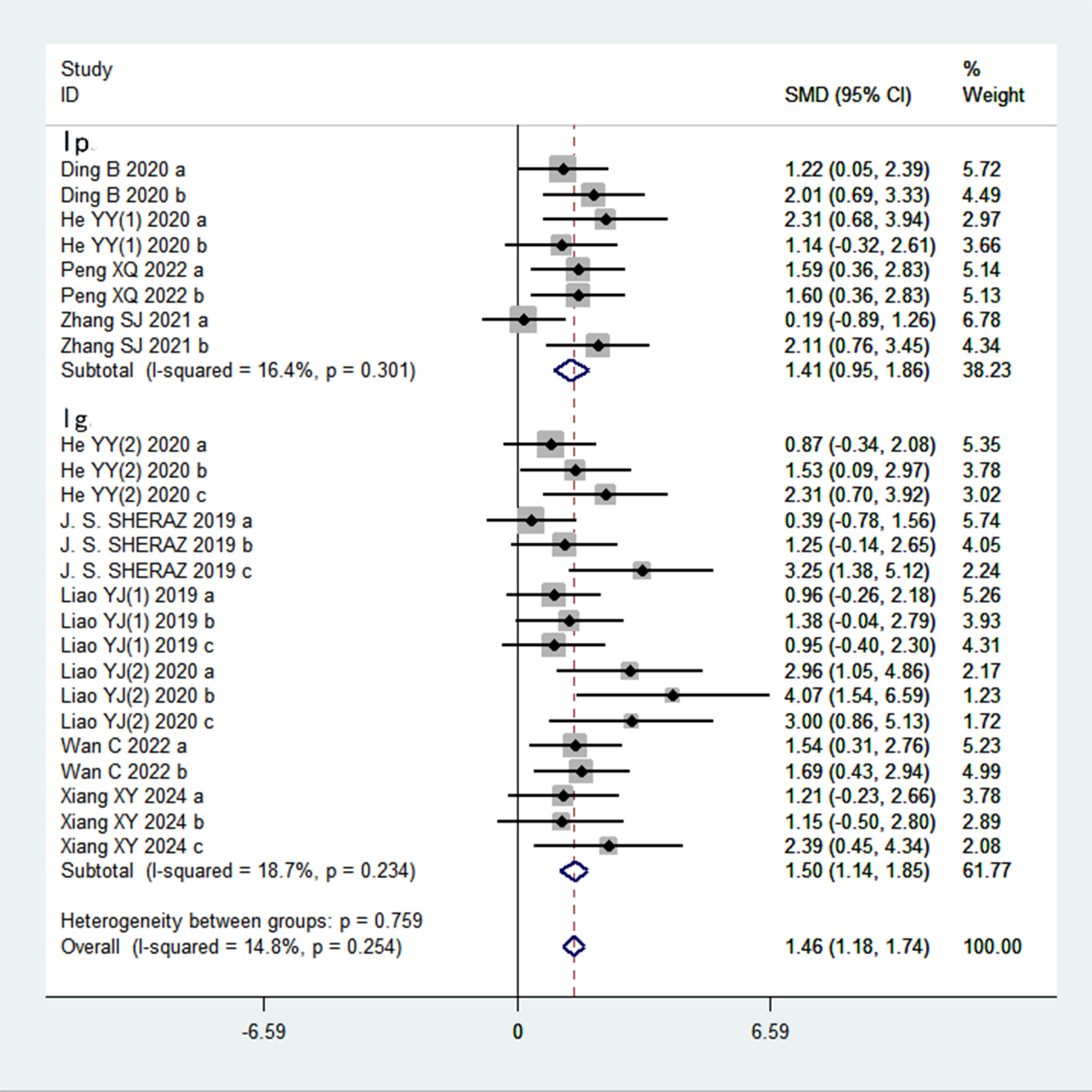


## Subgroup analysis of time in target quadrants after **tanshinones** intervention according to **the route of administration**


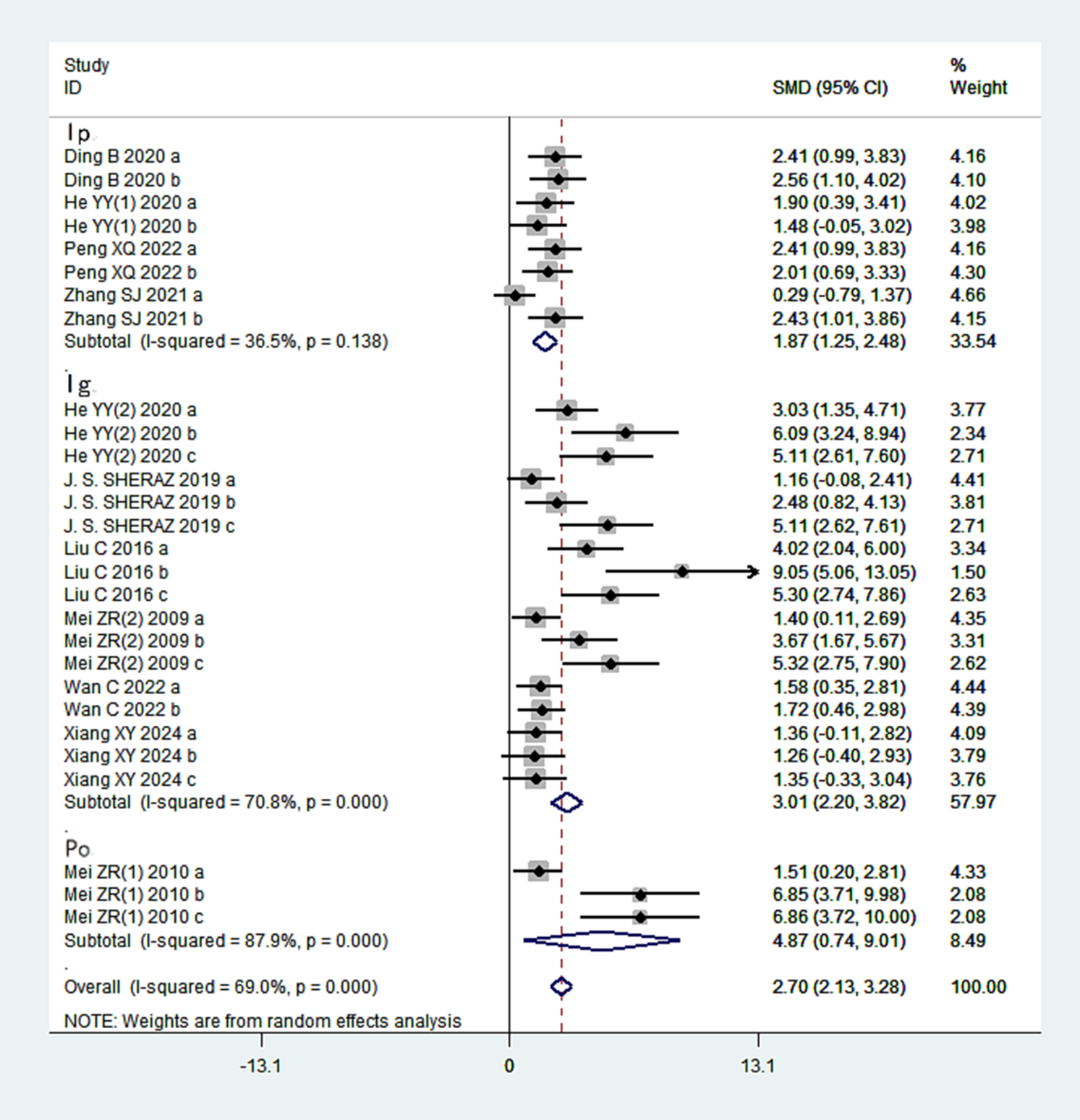


## Subgroup analysis of escape latency after doses of 20 mg or 25 mg of tanshinones intervention according to **the duration of the treatment**


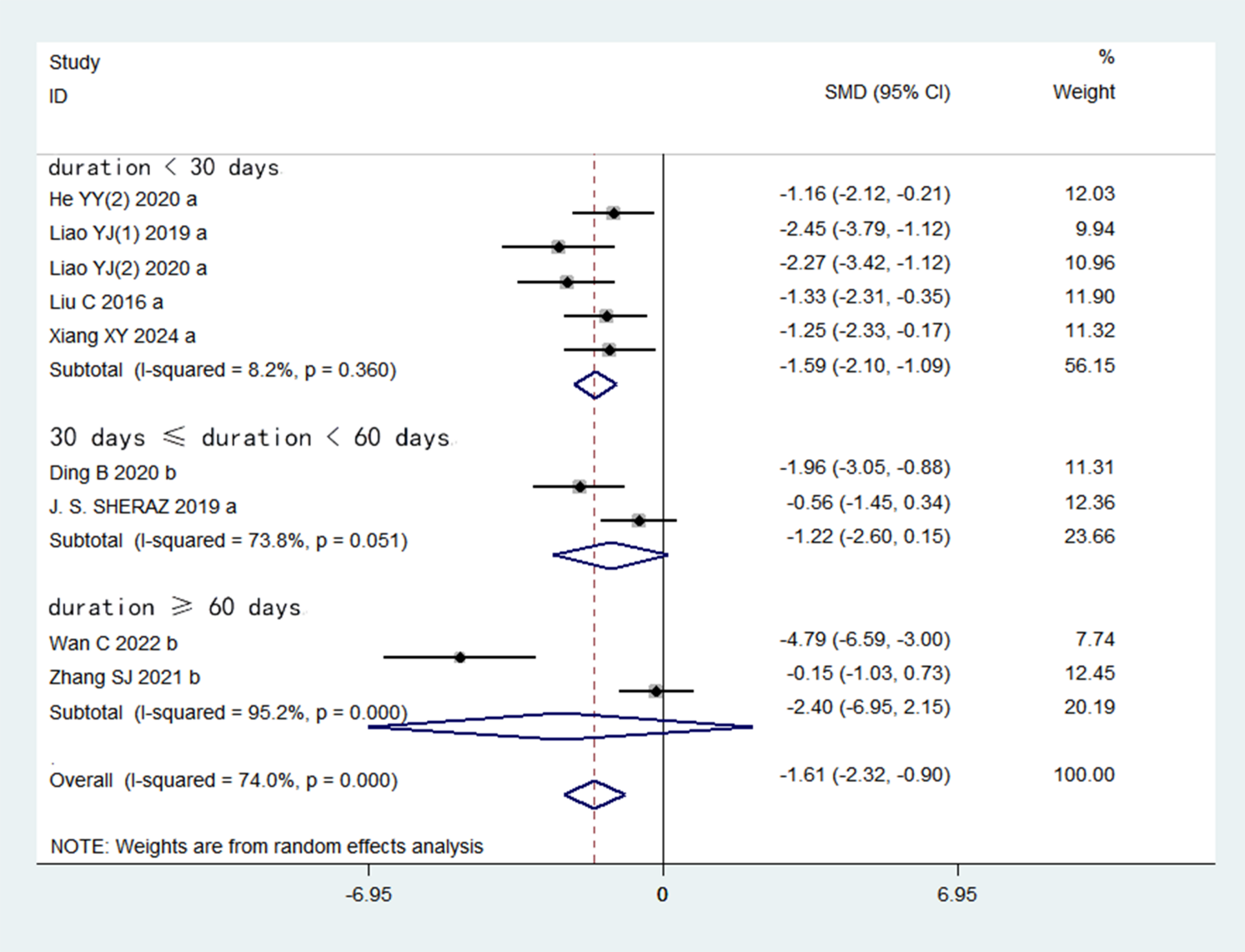


## Subgroup analysis of platform crossing times after doses of 20 mg or 25 mg of tanshinones intervention according to **the duration of the treatment**


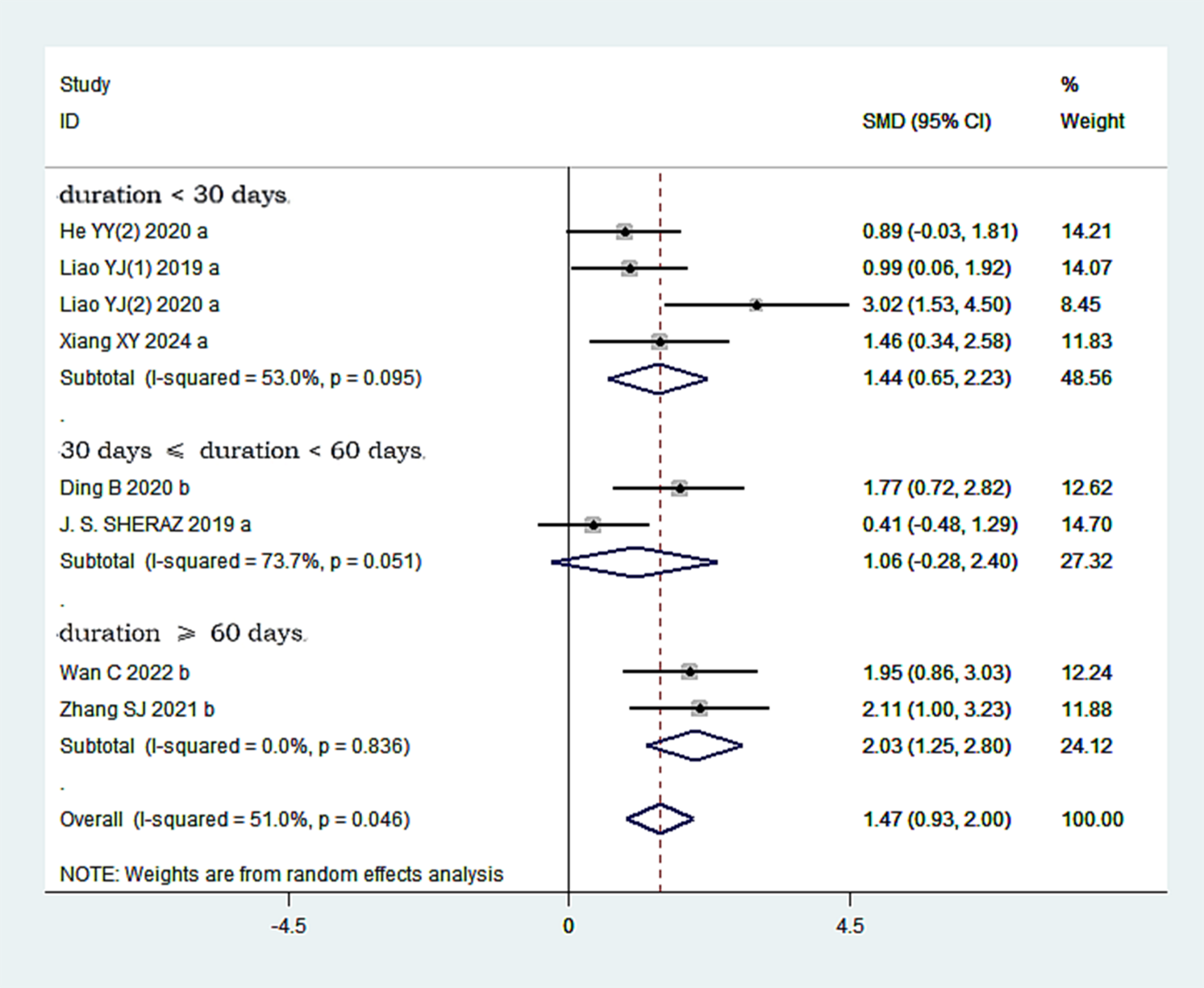


## Subgroup analysis of time in target quadrants after doses of 20 mg or 25 mg of tanshinones intervention according to **the duration of the treatment**


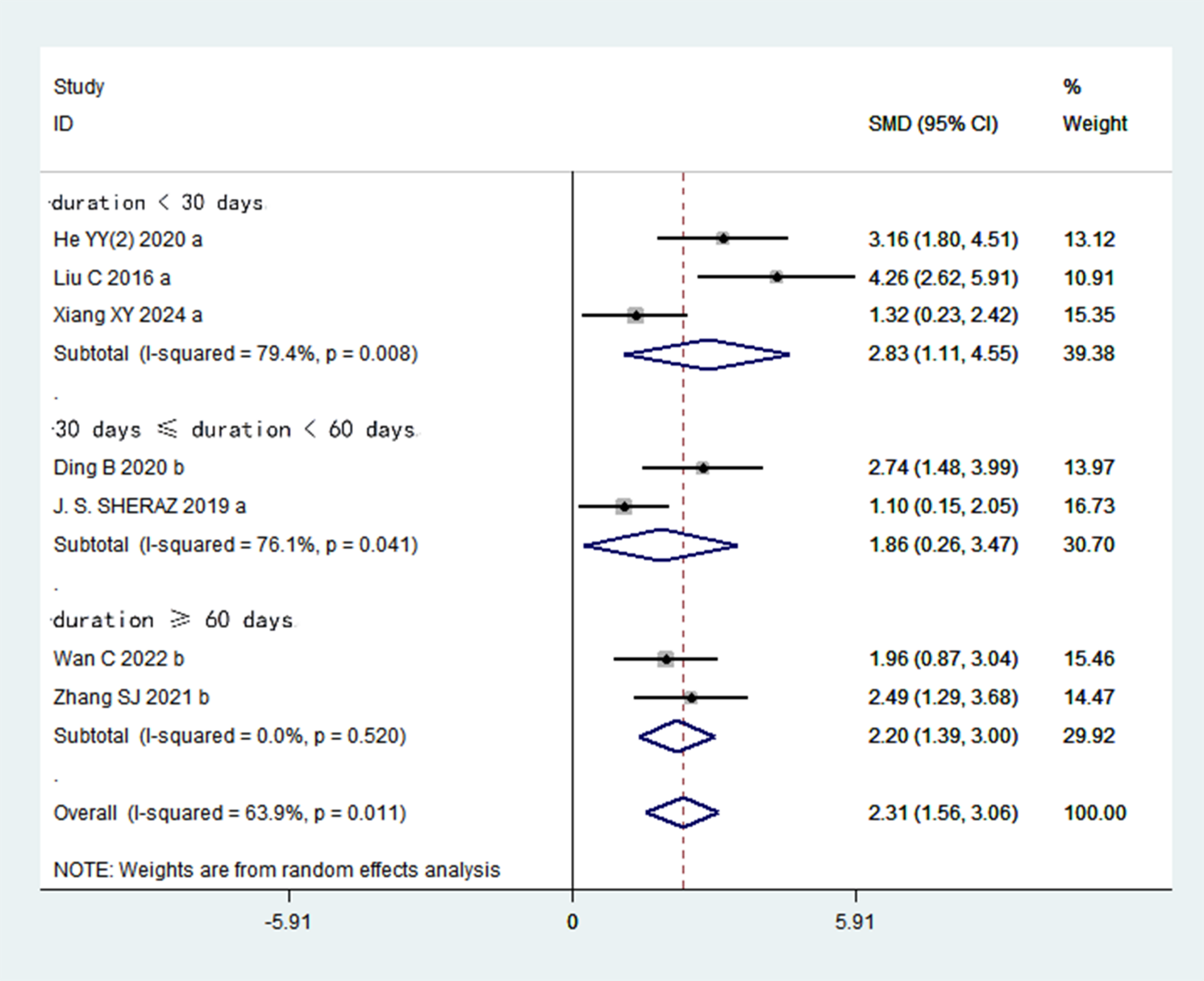


# Meta-regression analysis

## 2.1 Meta-regression of escape latency after doses of 20 mg or 25 mg of **tanshinones** intervention according to AD model

## Meta-analysis of platform crossing times after doses of 20 mg or 25 mg of **tanshinones** intervention according to AD model

## Meta-analysis of time in target quadrants after doses of 20 mg or 25 mg of **tanshinones** intervention according to AD model

##

## Meta-analysis of escape latency after doses of 15 mg or 20 mg of **tanshinones** intervention according to **the type of tanshinones**

## Meta-analysis of time in target quadrants after doses of 15 mg or 20 mg of **tanshinones** intervention according to **the type of tanshinones**

## Meta-analysis of escape latency after **tanshinones** intervention according to **the dosage of tanshinones**

## Meta-nalysis of platform crossing times after **tanshinones** intervention according to **the dosage of tanshinones**

##

## Meta-analysis of time in target quadrants after **tanshinones** intervention according to **the dosage of tanshinones**

## Meta-analysis of escape latency after **tanshinones** intervention according to **the route of administration**

## Meta-analysis of platform crossing times after **tanshinones** intervention according to **the route of administration**

## Meta-analysis of time in target quadrants after **tanshinones** intervention according to **the route of administration**

## Meta-analysis of escape latency after doses of 20 mg or 25 mg of tanshinones intervention according to **the duration of the treatment**

## Meta-analysis of platform crossing times after doses of 20 mg or 25 mg of tanshinones intervention according to **the duration of the treatment**

## Meta-analysis of time in target quadrants after doses of 20 mg or 25 mg of tanshinones intervention according to the duration of the treatment

# Sensitivity analysis

# 3.1 Sensitivity analysis of escape latency after tanshinones intervention


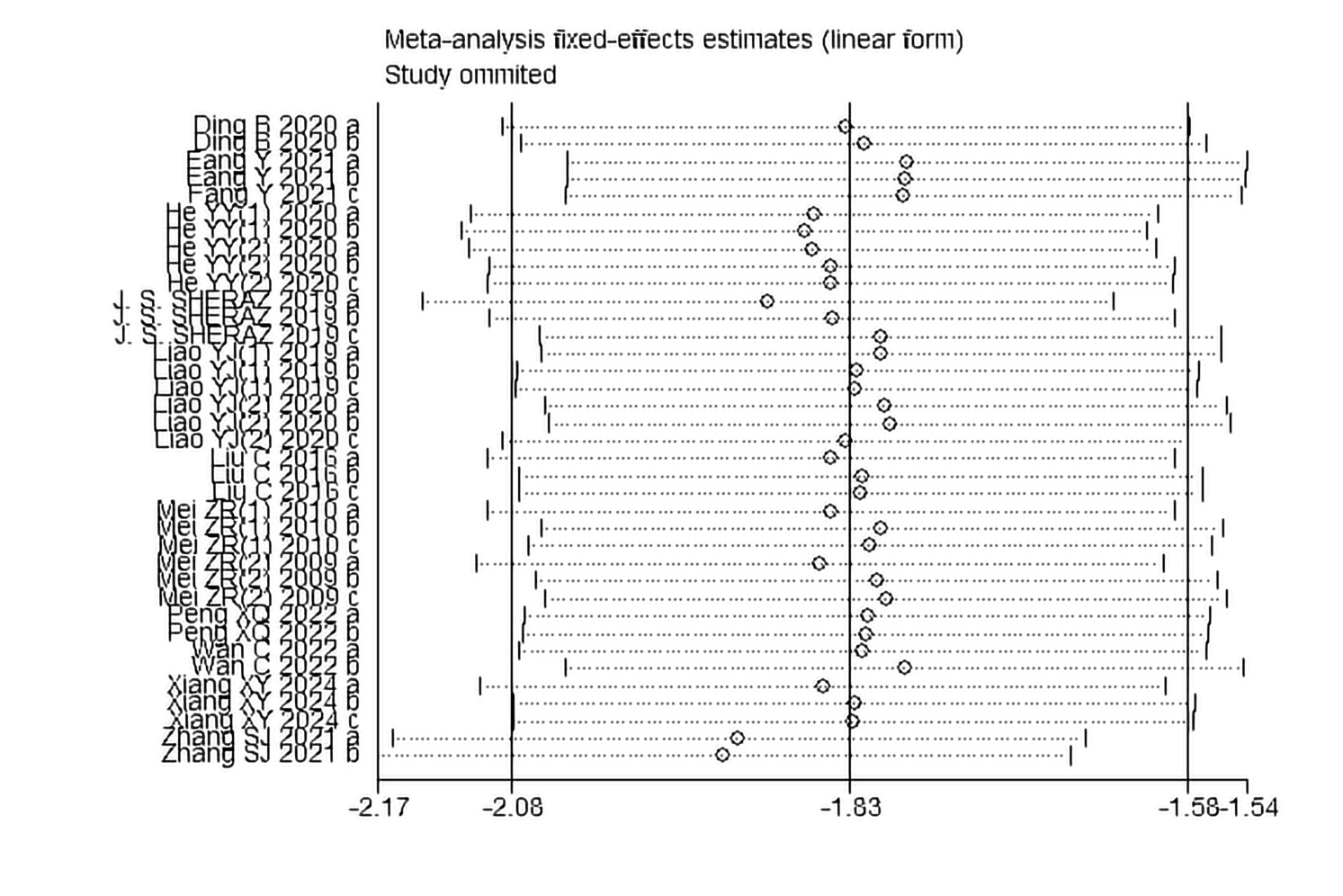


# 3.2 Sensitivity analysis of platform crossing times after tanshinones intervention


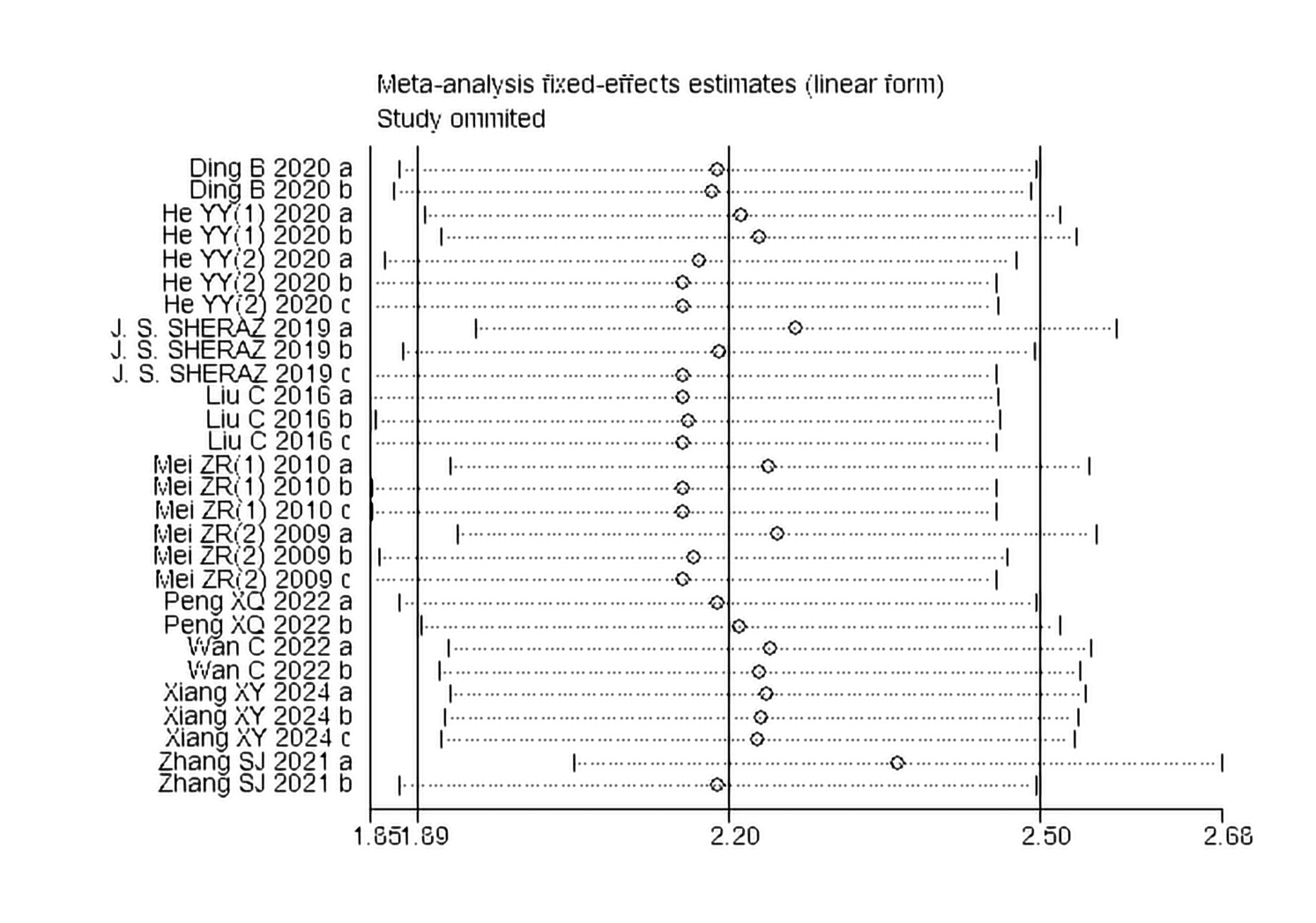


# 3.3 Sensitivity analysis of time in target quadrants after tanshinones intervention


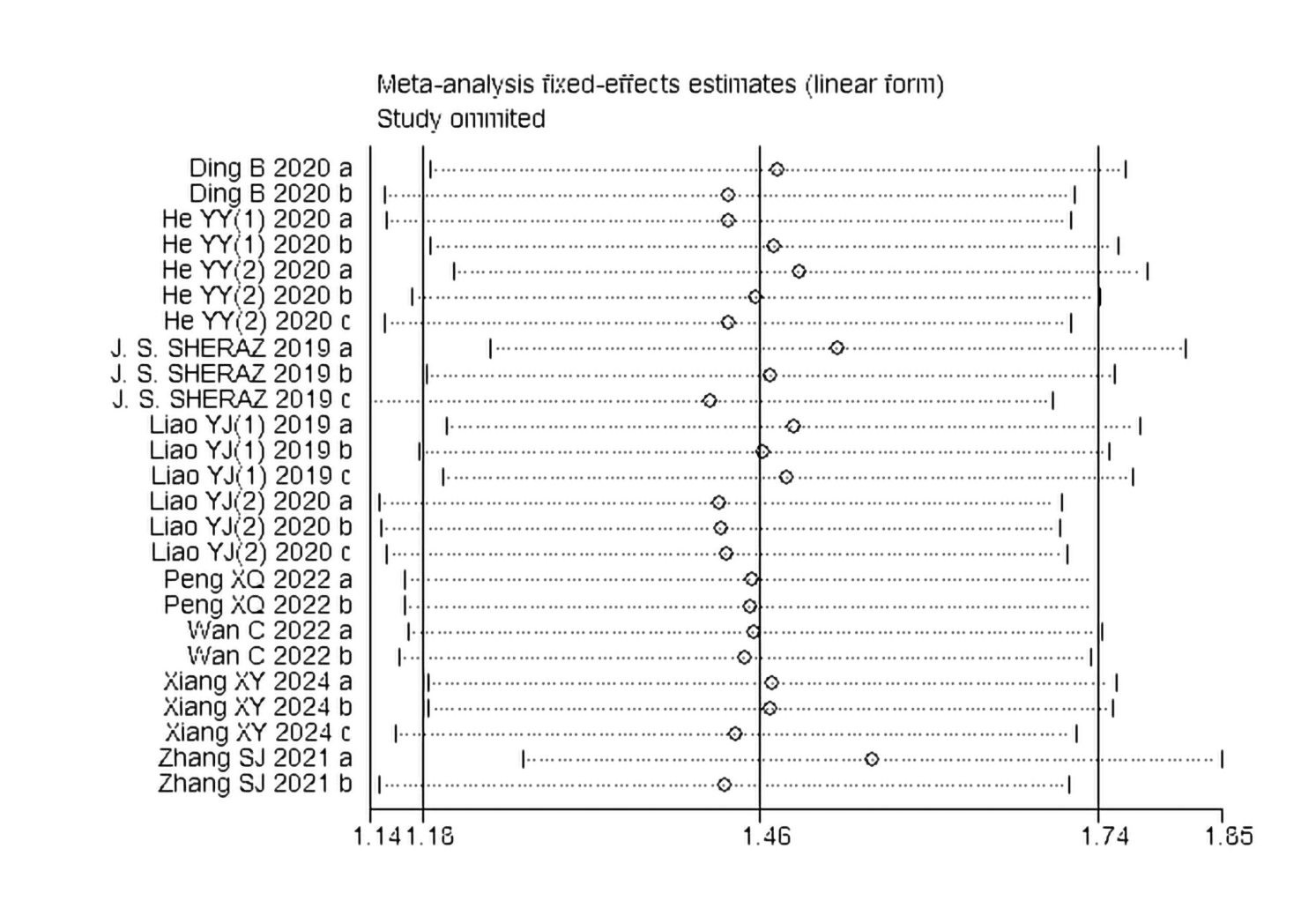

Supplement: Supplementary file 2 [file DataSheet2.docx]
